# ﻿Not (only) poison pies – *Hebeloma* (Agaricales, Hymenogastraceae) in Mexico

**DOI:** 10.3897/mycokeys.90.85267

**Published:** 2022-06-30

**Authors:** Ursula Eberhardt, Alejandro Kong, Adriana Montoya, Nicole Schütz, Peter Bartlett, Henry J. Beker

**Affiliations:** 1 Staatliches Museum für Naturkunde Stuttgart, Rosenstein 1, 70191 Stuttgart, Germany; 2 Centro de Investigación en Ciencias Biológicas, Universidad Autónoma de Tlaxcala, Km 10.5 carretera San Martín Texmelucan-Tlaxcala, San Felipe Ixtacuixtla, Tlaxcala, 90120, Mexico; 3 La Baraka, Gorse Hill Road, Virginia Water, Surrey GU25 4AP, United Kingdom; 4 Rue Père de Deken 19, B-1040 Bruxelles, Belgium; 5 Royal Holloway College, University of London, Egham, UK; 6 Plantentuin Meise, Nieuwelaan 38, B-1860 Meise, Belgium

**Keywords:** barcodes, Basidiomycota, ectomycorrhizal fungi, edible fungi, 3 new species, type studies

## Abstract

The species of *Hebeloma* have been little studied in Mexico, but have received attention as edibles and in trials to enhance production of edible fungi and tree growth through inoculation of seedlings with ectomycorrhizal fungi. Here we describe three new species of *Hebeloma* that are currently known only from Mexico. These species belong to separate sections of the genus: *H.ambustiterranum* is a member of H.sect.Hebeloma, *H.cohaerens* belongs to H.sect.Theobromina, while *H.magnicystidiatum* belongs to H.sect.Denudata. All three species were collected from subtropical pine-oak woodland; all records of *H.cohaerens* came from altitudes above 2500 m. *Hebelomaambustiterranum* is commonly sold in the local markets of Tlaxcala as a prized edible mushroom. An additional nine species are reported from Mexico, of which eight are new records for the country: *H.aanenii*, *H.eburneum*, *H.excedens*, *H.ingratum*, *H.neurophyllum*, *H.sordidulum*, *H.subaustrale* and *H.velutipes*. First modern descriptions of *H.neurophyllum* and *H.subaustrale*, originally described from the USA, are given here.

## ﻿Introduction

Arguably, the best recognized vernacular English name for the genus *Hebeloma* is poison pie, although this name is often reserved for *H.crustuliniforme*, and other species within the genus are qualified versions of this name, e.g. *H.mesophaeum* is the veiled poison pie and *H.pusillum* is the dwarf poison pie (https://www.britmycolsoc.org.uk/library/english-names, accessed 18 Nov 2021). The name poison pie suggests what is, certainly in Europe, believed to be true for all members of the genus: that they are poisonous, or even if they were not, all too easily mixed up with poisonous members of the genus. Collecting *Hebeloma* for human consumption is generally discouraged (Bresinsky and Besl 1990; Benjamin 1995).

In Mexico, the main interest in *Hebeloma* from the local community was either in the context of edibility (e.g., Montoya et al. 2008; Reyes-López et al. 2020) or with regard to the inoculation of trees of forest importance with ectomycorrhizal fungi (Pérez-Moreno et al. 2020 and references therein; Pérez-Moreno et al. 2021). A number of *Hebeloma* species were mentioned in these articles, including *H.alpinum*, *H.helodes*, *H.leucosarx* and *H.mesophaeum*.

We have not had the opportunity to examine the material used in the respective publications. Given the difficulty surrounding species concepts of this genus, the presence of these species in Mexico should be treated with caution. Both, with regard to the consumption of mushrooms and the inoculation of tree seedlings, it would be advantageous to have a clear understanding of the species involved and the morphological and molecular characters that define them to recognize or verify collections or strains.

To the best of our knowledge, *Hebeloma* are not included in commercial ectomycorrhizal fungi mixtures currently sold to enhance tree growth, but it is one of the few genera that have been used in numerous nursery trials and transplanting experiments (e.g., Castellano and Molina 1989; Barroetaveña and Rajchenberg 2005; Gagné et al. 2006; Oliveira et al. 2010 and see below). Owing to the difficulties delimiting and identifying *Hebeloma* species, members of the genus have often been treated as if they all shared the same ecological traits. This is clearly not the case (Beker et al. 2016).

From the taxonomic side, the *Hebeloma* of North America have been largely neglected since the work of Hesler and Smith in the 1970s and 1980s (Hesler 1977 and his unpublished manuscript on North American species of *Hebeloma*, Smith et al. 1983) and never extensively studied within Mexico. This lack of understanding of species concepts can be illustrated by reference to observation websites. For example, iNaturalist (https://www.inaturalist.org/observations?place_id=6793&taxon_id=192716 accessed on 12 March 2021) listed 41 *Hebeloma* observations for Mexico, but just six of these observations had species names attached: one was referred to *H.mesophaeum* and five were referred to *H.crustuliniforme*. Mushroom Observer (https://mushroomobserver.org/observer/advanced_search?q=1eMh6 accessed 12 March 2021) listed just eleven *Hebeloma* records from Mexico, none of which were identified to species level. The Global Biodiversity Information Facility GBIF.org (GBIF Occurrence Download https://doi.org/10.15468/dl.wd7f75 accessed 18 November 2021) gave 169 results for *Hebeloma* from Mexico, of which 60 were identified to species level: *H.alpinum* (1), *H.crustuliniforme* (17), *H.edurum* (1), *H.fastibile* (19), *H.mesophaeum* (16), *H.sacchariolens* (1) and *H.sinapizans* (5). MycoPortal (https://mycoportal.org/portal/collections/list.php accessed on 18 November 2021) gave 105 results for *Hebeloma* of Mexico. 100 of these records were from the National Herbarium of Mexico Fungal Collection (MEXU), four were from the Field Museum of Natural History (F) and one was from USDA, the United States National Fungus Collections (BPI). Of these 105 collections, 86 had no species name given, ten were identified as *H.fastibile*, five as *H.sinapizans*, three as *H.mesophaeum* and one as *H.sacchariolens*. There were, of course, overlaps between these databases, and one should be cautious of determinations given the historical confusion regarding species definitions, but all species records indicate just 6 species recorded: *H.alpinum*, *H.crustuliniforme*, *H.laterinum* (= *H.edurum*, *H.fastibile*), *H.mesophaeum*, *H.sacchariolens* and *H.sinapizans*.

Beker et al. (2016) published a monograph on *Hebeloma* of Europe to provide a new foundation for the understanding of species of this genus, on which future studies could be built. Although this monograph only addressed the genus within Europe, it has provided a base both morphologically and molecularly. Since the publication of that monograph, a number of papers have been published describing new species of *Hebeloma* as well as resurrecting long forgotten names that can now be confirmed as valid (e.g., Cripps et al. 2019; Eberhardt et al. 2020a, 2020b, 2021a, 2021b, 2022a, 2022b; Monedero and Alvarado 2020).

Within this paper, we present a list of *Hebeloma* species we have found during analysis of herbarium collections from Universidad Autónoma de Tlaxcala (TLXM). The 90 collections studied came from two principal areas in Chihuahua and Tlaxcala but also included a few collections from the regions of Mexico City and Puebla. Within this set, two species were rediscovered, *H.neurophyllum* (Atkinson 1909) and *H.subaustrale* (Murrill 1945), originally described from the US. The identifications were verified by morphological and molecular type studies. Three species new to science were discovered and are described below as *H.ambustiterranum*, *H.cohaerens* and *H.magnicystidiatum*. These species belong to separate sections of the genus and are described below.

## ﻿Materials and methods

**Figure 1.**
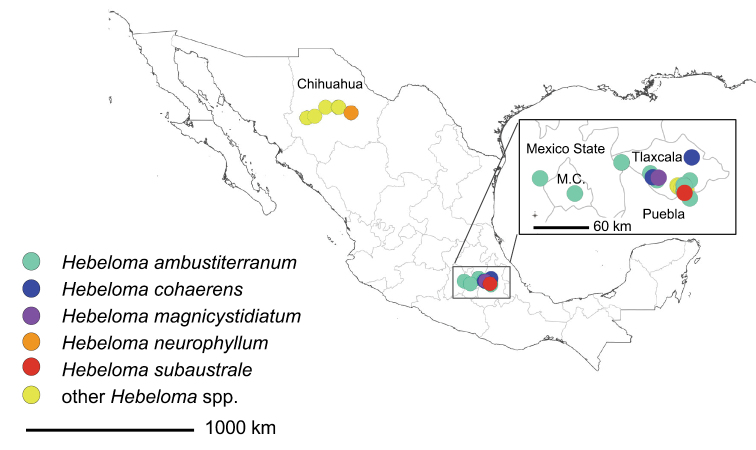
Collection sites of studied material. Scale bar 1000 km. The map was generated with QGIS version 3.16.15 using WGS84, EPDG: 4326 (QGIS Association, QGIS.org, 2022). Shapefiles were provided by the Database of Global Administrative Areas (GADM); Accessed April 2018 to March 2022.

All the material studied were dried specimens from the Universidad Autónoma de Tlaxcala (TLXM). The collections sites are shown in Fig. 1. These collections were compared to material collected for the *Hebeloma* project (Beker et al. 2016). Coordinates were obtained in the field by GPS or were approximated from the collection data. Approximations of elevations (m above sea level), where not recorded at time of collection, were deduced using Google Earth (Google Earth Pro Version 7.3.4.8248).

Sequences were obtained from the dried basidiomes by direct sequencing. At least the ITS (barcode) locus was generated for all Mexican collections and, in a number of cases, additional loci were sequenced. Internal transcribed spacer sequences were generated following methods detailed in Eberhardt (2012) and Cripps et al. (2019); *MCM7* (minichromosome maintenance complex component 7, partial) data following Eberhardt et al. (2016a); *RPB2* and *TEF1*a sequences following Eberhardt et al. (2021a); and sequences of two variable regions (V6 and V9) of the mitochondrial SSU followed Gonzalez and Labarère (1998). Sequencing was carried out by LGC Genomics (Berlin, Germany). Sequences were edited using Sequencher vs. 4.9 (Gene Codes Corp., Ann Arbor, Michigan). Newly generated sequences were accessioned to GenBank (ON167764–ON167898, ON168958–ON168966, ON202494–ON202614 and ON237944–ON237985), Suppl. material 1: Table S1 summarizes all sequences used in the analyses, including those previously published in the context of a number of publications (Eberhardt et al. 2009, 2013, 2015, 2016a, 2016b, 2021a, 2022a, 2022b; Eberhardt and Beker 2010; Beker et al. 2010, 2013, 2016; Schoch et al. 2012; Cripps et al. 2019).

Sequence alignments were done online in MAFFT using the E-INS-i option (Katoh et al. 2005, 2019) or locally with the “Mafft-globalpair” setting of MAFFT 7.471 (Katoh and Standley 2013). Alignments were done, viewed and reformatted in ALIVIEW 1.27 (Larsson 2014). Phylogenetic analyses (ML) were run in IQ-TREE (Nguyen et al. 2015) online (Trifinopoulos et al. 2016). Model selection (Kalyaanamoorthy et al. 2017) was done using the BIC criterion, including FreeRate models and merging partitions if possible (protein coding loci were originally partitioned according to position, coding and non-coding). Branch support was obtained through 1000 replicates of ultrafast bootstrap (ufb; Minh et al. 2013; Hoang et al. 2018) and SH-like approximate likelihood ratio tests (SH-aLRT; Guindon et al. 2010). Support values are given as (SH-aLRT [%]/ufb [%]), for SH-aLRT support ≥ 85% and ufb support ≥ 95%. Nexus files with alignments and trees, including all single locus trees, are available as Suppl. material 2.

Alignments were made for sections including new or rediscovered species, i.e., for H.sect.Hebeloma, H.sect.Naviculospora, H.sect.Theobromina and H.sect.Velutipes, including loci that were known to facilitate species recognition in the respective section (Beker et al. 2016). Sequences of types were included if available unless missing data (short sequences) had an adverse effect on the taxonomic resolution of the result. The selection of loci, additional species and taxa used for rooting was guided by previous results (Beker et al. 2016; Cripps et al. 2019; Eberhardt et al. 2021a, 2021b, 2022a, 2022b) – and by the loci that could be generated from the collections available. Prior to concatenation, single locus trees (see Suppl. material 2) were generated. Conflicts were detected using the principle by Kauff and Lutzoni (2002), assuming a conflict to be significant if two different relationships for the same set of taxa, one being monophyletic and the other non-monophyletic, were supported by SH-aLRT support ≥ 85% or ufb support ≥ 95%. Alignments of different loci were concatenated and analyzed, indicating branches with conflicting results from single locus analyses by dashed lines.

Distances between sequences were calculated from the alignments used for the ML analyses as p-distances with pairwise deletion of gaps in MegaX (Kumar et al. 2018; Stecher et al. 2020). The UNITE database (Kõljalg et al. 2013, 2020) and plutof (Abarenkov et al. 2010) were used for sequence searches, directly and via BLAST and for matching sequences to SH (species hypotheses).

Details of morphological analyses were provided in Beker et al. (2016). The amount of macroscopic detail available to us varied hugely from collection to collection as it was dependent on the detail provided by the collector. For recent collections where one of the authors was the collector, each specimen was photographed and observed both in the field when characters were still fresh, and later in the laboratory. Fresh basidiomes of each specimen were dried using a food dehydrator.

All microscopic analysis was carried out on dried material, using a Leica DMRZA2 microscope with a Leica DFC495 camera connected to a computer running Leica Application Suite (LAS) V4 software.

The basidiospores were first studied in Melzer’s reagent to assess the shape, degree of dextrinoidity, ornamentation and the degree of loosening of the perispore. For the assessment of the degrees of ornamentation (O0, O1, O2, O3, O4), of the loosening perispore (P0, P1, P2, P3) and for the dextrinoidity (D0, D1, D2, D3, D4), we used Beker et al. (2016) and Vesterholt (2005). A number of photographs were taken of the basidiospores at ×500 and ×1600, which were then measured using the LAS software. For each collection, wherever possible, at least 50 basidiospores were measured in Melzer’s reagent, excluding the apiculus. As discussed in Beker et al. (2016), the difference in *Hebeloma* basidiospore size from dried material, measured in Melzer’s reagent and 5% KOH, is negligible. The maximum length and width of each spore was measured, and its Q value (ratio of length to width) calculated. Average length, width, and Q value were calculated and recorded alongside the median, standard deviation, and 5% and 95% percentiles.

The material was then examined in 5% KOH. Photographs were taken of the basidiospores and also of the cheilocystidia (and pleurocystidia if any were present) and basidia at ×500 and ×1000. Because of the complex shapes of the cheilocystidia four measurements were made: length, width at apex (A), width at narrowest point in central region (M), and maximum width in lower half (B). The measurements were given in this order, and an average value was calculated for each of these measurements. The average width of the cheilocystidium in the vicinity of the apex appears to be an important character in the separation of species within *Hebeloma* (Vesterholt 2005). It is also important, when determining this average width near the apex, not to be selective with regard to the cystidia chosen for measurement. To determine the average width at the apex, about 100 cheilocystidia were measured on the lamella edge. For other measurements, some 20 cheilocystidia, separated from the lamella edge, were measured from each collection. For each cheilocystidium the ratios A/M, A/B, and B/M were calculated and averaged across all cheilocystidia measured. For all other details with regard to our methodology, see Beker et al. (2016).

Each collection studied has a database record number associated with that collection (beginning HJB); we give these numbers as we intend to make the database publicly available. If no other herbarium abbreviation or herbarium accession number is given, the HJB number is also the collection number within H.J. Beker’s herbarium.

Species were identified considering morphological and molecular data. In cases in which molecular data were not conclusive (as e.g., for *H.eburneum* and *H.velutipes*, or could not be obtained, as for the type of *H.subaustrale*), species identification followed morphology. For species not discussed in detail here, please refer to species descriptions in Beker et al. (2016) and Eberhardt et al. (2021a, 2022a).

## ﻿Results

It appears that all of the species found in our sample, other than *Hebelomamesophaeum*, are new species records for Mexico. Fig. 1 shows the distribution of these fungal collections in Mexico; Suppl. material 1: Table S1 lists all collections utilized during this study, including those not specifically discussed in the Taxonomy part.

**Figure 2.**
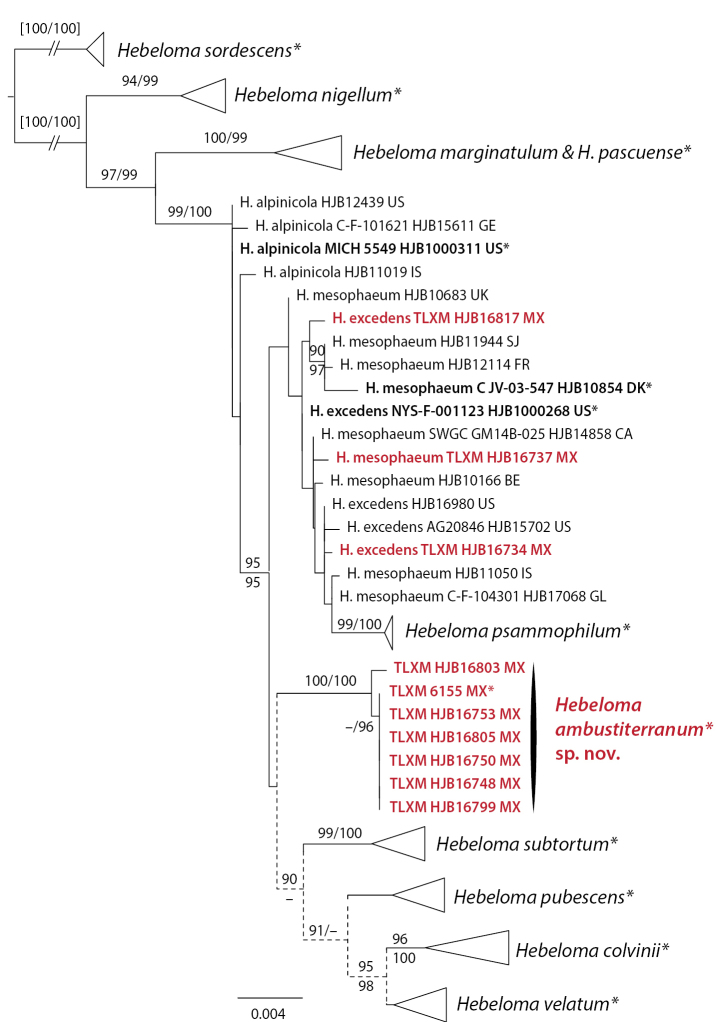
ML topology of concatenated ITS, *RPB2* and *TEF1a* sequences of Hebelomasect.Hebeloma. Branch support was obtained through 1000 replicates of SH-like approximate likelihood ratio tests and ultrafast bootstrap annotated SH-aLRT/ufb at the branches for ≥ 85% SH-aLRT and ≥ 95% for ufb support. Dotted lines indicate parts of the tree where conflicts between single locus results were observed. *Hebelomasordescens* (H.sect.Hebeloma) was used for rooting. Collections indicated with * are types; clade names indicated by * include type sequences. Collections and species names in red refer to Mexican material.

The analysis of taxa from H.sect.Hebeloma (from Mexico *H.ambustiterraneum*, *H.excedens* and *H.mesophaeum*) included ITS, *RPB2* and *Tef1a* data, and 67 collections from 13 species. *Hebelomasordescens* (H.sect.Hebeloma) was used for rooting. *Hebelomaambustiterranum* was monophyletic in all single locus results and received support in ITS (100/100%) and *RPB2* (85/98%). Conflicts between ITS and the other two loci were observed in relation to the position of *H.pubescens* (p.p.) and *H.subtortum* (ITS with *H.excedens*, *H.mesophaeum* and *H.psammophilum*; *RPB2* and *TEF1a* with *H.colvinii* and *H.velatum* [= *H.dunense*, Eberhardt et al. 2022a] and within *H.pubescens* [collection HJB12057]). Neither of these conflicts were considered relevant in the current context. The concatenated alignment spanned 2205 positions. The clade of *H.ambustiterranum* (Fig. 2) received full (100/100%) support. This result supported morphology in that *H.ambustiterranum* is a good species new to science. Neither *H.excedens* nor *H.mesophaeum* were resolved (Fig. 2); the Mexican collections of these two species were placed among other members of *H.excedens* and *H.mesophaeum*.

**Figure 3.**
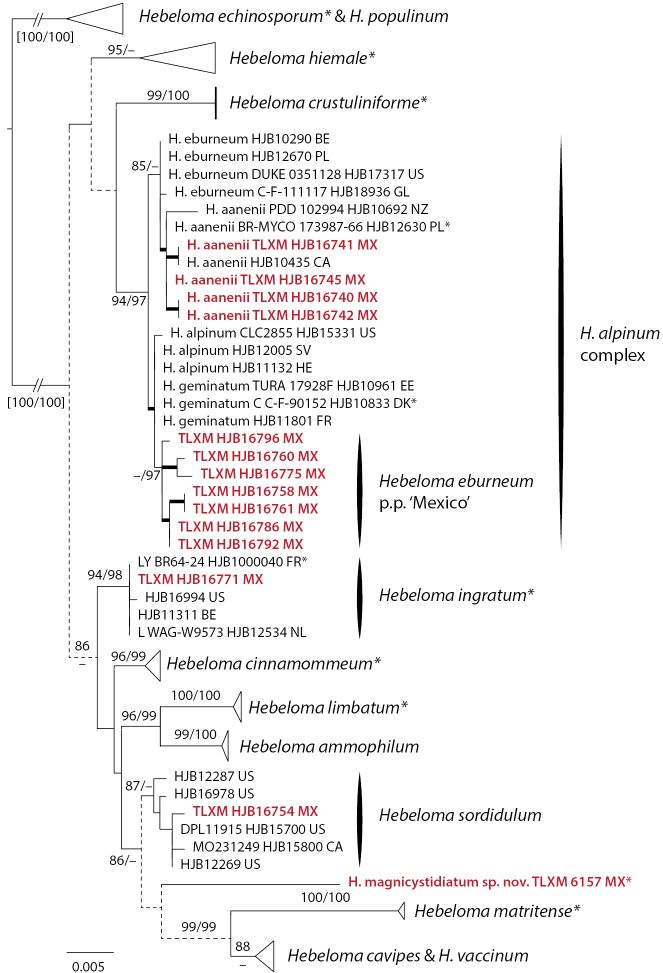
ML topology of concatenated ITS, mitSSU V6 and V9 sequences of Hebelomasect.Denudata. Branch support was obtained through 1000 replicates of SH-like approximate likelihood ratio tests and ultrafast bootstrap annotated SH-aLRT/ufb at the branches for ≥ 85% SH-aLRT and ≥ 95% for ufb support or by thick lines in the case that at least one of the support values is equal to or exceeds the limits. Dotted lines indicate parts of the tree where conflicts between single locus results were observed. *Hebelomaechinosporum* and *H.populinum* (H.subsect.Echinospora of H.sect.Denudata) were used for rooting. Collections indicated with * are types; clade names indicated by * include type sequences. Collections in red refer to Mexican material.

The analysis for H.sect.Denudata (in Mexico *H.aanenii*, *H.eburneum*, *H.ingratum*, *H.magnicystidiatum* and *H.sordidulum*) was based on ITS, mitSSU V6 and V9 of 78 collections from 17 species. *Hebelomaechinosporum* and *H.populinum* (H.sect.Denudata, subsect. Echinospora) were used for rooting. In the ITS tree, *H.magnicystidiatum* was part of the *H.sordidulum* clade (90/–%), which was included in a weakly supported clade (90/–%) with all other members of H.subsect.Clepsydroida considered in the analysis. Neither of the mitSSU results contradicted this relationship with any support, but there were conflicts between the ITS and mitSSU results and between the two mitSSU results in relation to the limits of the subsections and the relationship of *H.hiemale* (H.subsect.Hiemalia) and H.subsect.Clepsydroida and H.subsect.Crustuliniformia. In spite of this, the alignments were concatenated. The resulting phylogenetic hypothesis (Fig. 3) showed *H.magnicystidiatum* outside the clade of *H.sordidulum* (which was only weakly supported, 85/–%), but on a relatively long branch, thus supporting morphology that *H.magnicystidiatum* is a separate species. Because of existing conflicts, molecular data could not resolve the position of *H.magnicystidiatum* in any of *H.* subsects. *Clepsydroida*, *Crustuliniformia* or *Hiemalia*.

The Mexican collections of *H.aanenii* clustered with their conspecifics from other countries, while the Mexican collections of *H.eburneum* were not in the same clade as *H.eburneum* collections from other countries, both clades received some support, one by ufb, the other by SH-aLRT (see Fig. 3). The only single locus tree showing a Mexican *H.eburneum* clade is mitSSU V6 (86/95% support). Both *H.eburneum* clades were, as well as *H.aanenii*, in what Beker et al. (2016) termed the *H.alpinum*-complex (94/97% support). The Mexican collection of *H.ingratum* was included in the *H.ingratum* clade (93/98% support); the Mexican collection of *H.sordidulum* was included in the respective species clade, which only received 87/– support.

**Figure 4.**
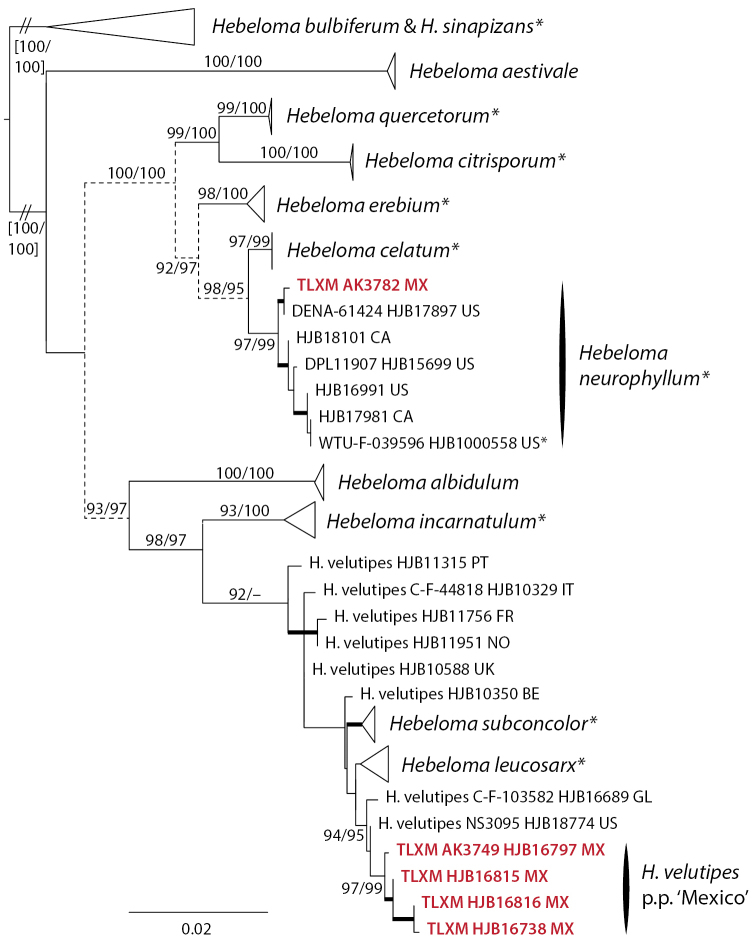
ML topology of concatenated ITS, *RPB2* and *TEF1a* and mitSSU V6 sequences of Hebelomasect.Velutipes. Branch support was obtained through 1000 replicates of SH-like approximate likelihood ratio tests and ultrafast bootstrap annotated SH-aLRT/ufb at the branches for ≥ 85% SH-aLRT and ≥ 95% for ufb support or by thick lines in the case that at least one of the support values is equal to or exceeds the limits. Dotted lines indicate parts of the tree where conflicts between single locus results were observed. *Hebelomabulbiferum* and *H.sinapizans* (H.sect.Sinapizantia) were used for rooting. Collections indicated with * are types; clade names indicated by * include type sequences. Collections in red refer to Mexican material.

The analysis for H.sect.Velutipes (in Mexico *H.neurophyllum* and *H.velutipes*) was based on ITS, *RPB2*, *TEF1a* and mitSSU V6 of 59 collections from 12 species. *Hebelomabulbiferum* and *H.sinapizans* (H.sect.Sinapizantia) were used for rooting. *Hebelomaneurophyllum* received good support (95/95%) in the ITS result, and is paraphyletic in relation to *H.erebium* in the *RPB2* and *TEF1a* results, and in relation to *H.celatum* in the mitSSU V6 result. In spite of a number of conflicts concerning interspecific relationships within H.sect.Velutipes—intraspecific conflicts were not detected—the different single locus alignments were concatenated. The alignment included 2670 positions. In the analysis of the concatenated dataset (Fig. 4), *H.neurophyllum* was well supported (97/99%), as were *H.celatum* (97/ 99%) and *H.erebium* (98/100%). Thus, molecular data as well as morphological characters (see below) supported *H.neurophyllum* as a good species.

*Hebelomavelutipes* was paraphyletic in relation to the other member species of the *H.velutipes* complex clade (*H.incarnatulum*, *H.leucosarx* and *H.subconcolor*). The position of the Mexican collections of *H.velutipes* in a separate clade (97/99%) was only supported by the mitSSU V6 data.

**Figure 5.**
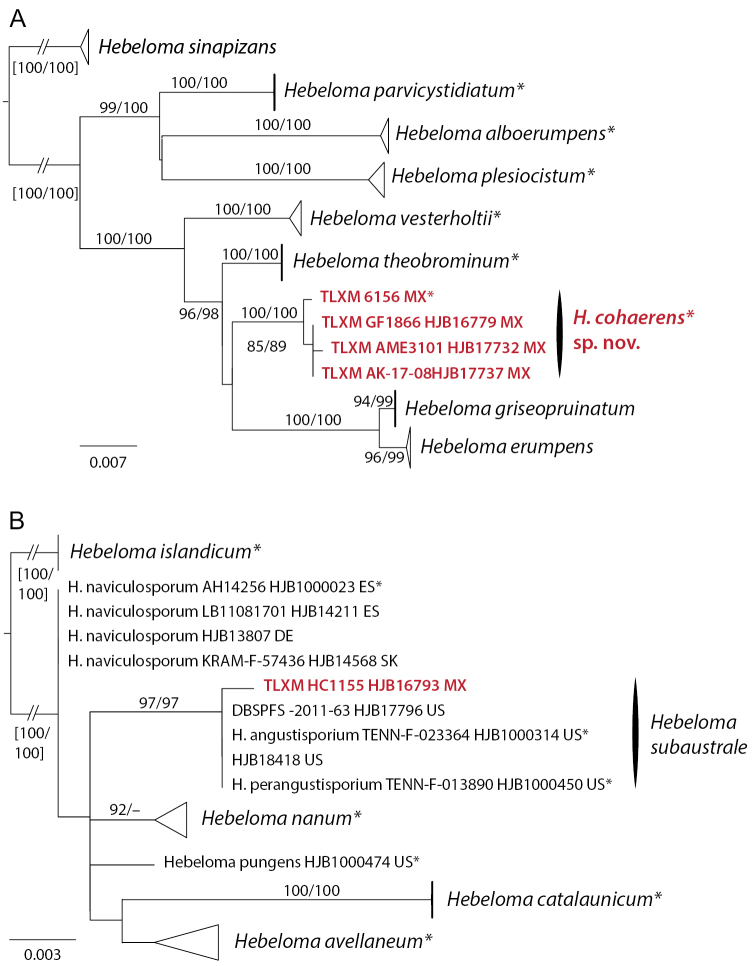
ML topologies with branch support obtained through 1000 replicates of SH-like approximate likelihood ratio tests and ultrafast bootstrap annotated SH-aLRT/ufb at the branches for ≥ 85% SH-aLRT and ≥ 95% for ufb support. Collections indicated with * are types; clade names indicated by * include type sequences. Collections in red refer to Mexican material **A** concatenated ITS, *MCM7* and *RPB2* sequences of Hebelomasect.Theobromina, rooted with *H.sinapizans* (H.sect.Sinapizantia) **B** ITS sequences of H.sect.Naviculospora, rooted with *H.islandicum* (H.sect.Naviculospora).

The analysis for H.sect.Theobromina (in Mexico *H.cohaerens*) was based on ITS, *MCM7* and *RPB2* of 32 collections from nine species. *Hebelomasinapizans* was used for rooting. *Hebelomacohaerens* was supported by all three single locus analyses (96–97/95–100%) and received full (100/100%) support in the analysis of the concatenated data (2152 bp) (Fig. 5A). No conflicts were found between the single locus results. Thus, both molecular results and morphology supported *H.cohaerens* as a new species.

The analysis for H.sect.Naviculospora (in Mexico *H.subaustrale*) was based on the ITS of 24 collections of eight species and included 703 positions. *Hebelomaislandicum*, provisionally placed by Beker et al. (2016) in H.sect.Naviculospora to avoid creating a monospecific section for the species, was used for rooting. Holotype sequences generated by P.B. Matheny and A.D. Wolfenbarger of *H.angustisporium* (NR_119890, Schoch et al. 2014) and of *H.perangustisporium* (HQ179680, unpublished, submitted 23 Aug 2010) and by H. Gordon of *H.pungens* (MW412387, unpublished, submitted 28 Dec 2020) were identical or almost identical with our sequences but had shorter read length in the analyzed region. Thus, only the sequences generated by us were considered in the analysis. The holotype sequences of *H.angustisporium* and *H.perangustisporium*, as well as three morphologically matching collections formed a clade supported by 97/97% among all other recognized members of H.sect.Naviculospora. Morphologically, the *H.angustisporium* and *H.perangustisporium* agree with *H.subaustrale*, which is the oldest of the three names. Thus, although no sequence data could be obtained for the type of *H.subaustrale*, the clade is referred to as *H.subaustrale* in Fig. 5B, and *H.subaustrale* is accepted and described below.

### ﻿Taxonomy

For species described from Europe please refer to Beker et al. (2016); for *H.excedens* and *H.sordidulum* to Eberhardt et al. (2022a) and for *H.excedens* also to Cripps et al. (2019).

#### ﻿ Hebeloma ambustiterranum

Taxon classificationFungiAgaricalesHymenogastraceae

A. Kong & Beker, sp. nov.

F8B2C92E-CDE6-5C79-AF5D-7B540AC562D9

842826

Figs 6 , 7

##### Type.

Mexico. Tlaxcala: La Malinche National Park, 19.2749°N, 97.9825°W, alt. approx. 2800 m, on burnt soil in coniferous woodland under *Pinusmontezumae* and *P.teocote*, 8 Jul 2017, H.J. Beker HJB16802 (holotype TLXM 6155; isotype BR 5020224874626V); GenBank ITS ON202501.

##### Diagnosis.

The small ellipsoid, non-dextrinoid, almost smooth basidiospores (on average 8.0–10.2 × 5.6–6.5 µm) and at least 50 full length lamellae distinguish this species from all other known North American *Hebeloma* species and the ITS sequence differentiates this species from all other known species, worldwide.

##### Etymology.

From *ambustus* (Latin adj.) meaning scorched, *terra* (Latin n.) meaning soil and the Latin suffix -*anum* indicating position to indicate growing on scorched soil. In Mexico, the local people burn the ground in the pine forests to encourage the growth of this mushroom, which they regard as an excellent edible mushroom. The local people refer to it in Nahuatl as the xolete de ocoxal (or ocoxalnanacatl), the mushroom of the pine needles from Chamusquinero, meaning from burnt ground.

##### Description.

Pileus (12) 16–45 (52) mm diameter, usually umbonate or subumbonate, rarely convex or applanate; margin usually entire, sometimes involute particularly when young, often with remains of the universal veil, occasionally spotting, not hygrophanous; usually almost unicolored with color at center usually cream to ochraceous or clay-buff but may occasionally be darker, honey to sepia or umber, usually a little paler at the margin. Lamellae emarginate, white, cream to brown, with a weak white fimbriate edge sometimes visible and without droplets, number of full-length lamellae 50–74. Stipe (23) 24–60 (75) mm long, 3–8 (10) mm diameter at median, cylindrical, surface cream, ivory to pale brown but occasionally discoloring from the base upwards, sometimes strongly, fibrillose, at apex pruinose; base with white mycelium. Partial veil present on young specimens, whitish at first, before basidiospores mature, and often clear fibrils remaining on the stipe and pileus. Context in pileus white to cream, firm, in stipe stuffed, becoming hollow with age; taste not recorded, smell occasionally odorless but usually raphanoid, sometimes strongly so or with cacao components. Spore deposit color clay-buff.

Basidiospores based on n = 146 spores of the holotype, 5% to 95% percentile range 7.7–9.8 × 5.5–7.0 µm, with median 8.9 × 5.9 µm and av. 8.9 × 6.0 µm with S.D. length 0.68 µm and width 0.44 µm; Q value 5% to 95% percentile range 1.25–1.63, with median 1.48 and av. 1.47 with S.D. 0.11; spore size based on 33 collections medians 7.8–10.3 × 5.5–6.4 µm and av. 8.0–10.2 × 5.6–6.5 µm with av. S.D. length 0.61 µm and width 0.35 µm, av. Q 1.36 –1.61, ellipsoid or ovoid, with small apiculus, apex round or subacute, with a distinct thinning of the apical wall, guttulate with one or sometimes more oily drops, usually almost smooth even under immersion, with perispore not loosening, almost totally non-dextrinoid with just an indistinct brownish tint in Melzer’s reagent (O1; P0; D1); pale yellow to brown in KOH. Basidia 25–34 × 6–8 µm, with av. Q 3.7–4.4, cylindrical to clavate, hyaline, 4-spored. Cheilocystidium width near apex holotype 5% to 95% percentile range 3.5–5.3 µm, with median 4.3 µm and av. 4.3 µm with S.D. 0.63 µm; across 33 collections median 4.1–5.4 µm and av. 4.1–5.1 µm; examining approx. 20 selected cheilocystidia of each of the 33 collections yields a range for the avs. of 35–55 × 4.1–5.1 × 4.2–5.1 × 7.1–9.9 µm and 35 × 4.3 × 4.2 × 7.3 µm av. for holotype; av. ratios A/M: 0.96–1.15, A/B: 0.51–0.70, B/M: 1.55–2.31, mainly swollen in the lower half, some ventricose or lageniform, often with one or two septa, rarely geniculate or with some thickening of the median wall, hyaline. Pleurocystidia absent. Caulocystidia similar to cheilocystidia but more cylindrical and larger, up to 140 μm. Pileipellis an ixocutis; epicutis up to 100 µm thick, with gelatinized, often encrusted hyphae up to 6 µm wide; subcutis yellow and the trama below the cutis made up of cylindrical or occasionally ellipsoid cells up to 14 µm wide. Clamp connections present throughout the basidiome.

##### Ecology and distribution.

In temperate coniferous woodlands on burnt ground with *Pinus* and *Quercus*. Growth habit usually scattered, rarely solitary or caespitose. To date, all collections of *Hebelomaambustiterranum* recorded from Mexico at latitudes between 19°N and 20°N and altitudes above 2000 m.

##### Additional collections examined.

Mexico. **Mexico City**: Municipality of Milpa Alta, approx. 19.1942°N, 99.0267°W, alt. approx. 2400 m, 4 Jul 2011, R. Vanegas-Enriquez (TLXM RVE042, HJB17734). Municipality of Milpa Alta, approx. 19.1942°N, 99.0267°W, alt. approx. 2400 m, 16 Jul 2011, R. Vanegas-Enriquez (TLXM RVE049, HJB17735). Municipality of Milpa Alta, approx. 19.1942°N, 99.0267°W, alt. approx. 2400 m, 21 May 2013, A.C. López (TLXM ACL-MA-085, HJB17736). **Puebla**: Municipality of Acajete, La Malinche National Park, north of Santa Isabel Tepetzala, approx. 19.1471°N, 97.924°W, alt. approx. 2600 m, on soil in woodland under *Pinus* sp., 15 Jul 1998, R. Reyes-Lopez (TLXM RL1-01, HJB16780). Municipality of Acajete, La Malinche National Park, 4 km north of Santa Isabel Tepetzala, approx. 19.1471°N, 97.9239°W, alt. approx. 2600 m, on soil in woodland under *Pinus* sp., 29 Jul 1998, R. Reyes-López (TLXM RL2-7, HJB16765). **Tlaxcala**: La Malinche National Park, 19.2742°N, 97.9833°W, alt. approx. 2850 m), on burnt soil in coniferous woodland under *Pinusmontezumae* and *Pinusteocote*, 8 Jul 2017, Forayer (TLXM HJB16799). La Malinche National Park, 19.2744°N, 97.9831°W, alt. approx. 2850 m, on burnt soil in coniferous woodland under *Pinusmontezumae* and *Pinusteocote*, 8 Jul 2017, L. Davies (TLXM HJB16800). La Malinche National Park, 19.2743°N, 97.9829°W, alt. approx. 2840 m 8 Jul 2017, L. Davies (TLXM HJB16801), on burnt soil in coniferous woodland under *Pinusmontezumae* and *Pinusteocote*. La Malinche National Park, 19.2749°N, 97.9820°W, alt. approx. 2830 m, 8 Jul. 2017, A. Montoya-Esquivel, A. Kong (TLXM HJB16803), on burnt soil in coniferous woodland under *Pinusmontezumae* and *Pinusteocote*. La Malinche National Park, 19.2752°N, 97.9820°W, alt. approx. 2830 m, on burnt soil in coniferous woodland under *Pinusmontezumae* and *Pinusteocote*, 8 Jul 2017, A. Kong (TLXM HJB16804). La Malinche National Park, 19.2753°N, 97.9823°W, alt. approx. 2830 m, on burnt soil in coniferous woodland under *Pinusmontezumae* and *Pinusteocote*, 8 Jul. 2017, A. Montoya-Esquivel, A. Kong (TLXM HJB16805). La Malinche National Park, 19.2751°N, 97.9825°W, alt. approx. 2830 m, on burnt soil in coniferous woodland under *Pinusmontezumae* and *Pinusteocote*, 8 Jul 2017, A. Montoya-Esquivel, A. Kong (TLXM HJB16806). La Malinche National Park, 19.2754°N, 97.9824°W, alt. approx. 2830 m, on burnt soil in coniferous woodland under *Pinusmontezumae* and *Pinusteocote*, 8 Jul 2017, H.J. Beker (TLXM HJB16807). La Malinche National Park, 19.2755°N, 97.983°W, alt. approx. 2830 m, on burnt soil in coniferous woodland under *Pinusmontezumae* and *Pinusteocote*, 8 Jul 2017, A. Montoya-Esquivel, A. Kong (TLXM HJB16808). La Malinche National Park, 19.2652°N, 97.9744°W, alt. approx. 2825 m, on soil in coniferous woodland ditch under *Pinusteocote*, 9 Jul 2017, A. Montoya-Esquivel (TLXM HJB16818). Municipality of Huamantla, La Malinche National Park, Los Pilares, approx. 19.3184°N, 97.9233°W, alt. approx. 2500 m, on soil in woodland under *Pinus* sp., 2 Aug 1991, A. Montoya-Esquivel (TLXM AME1048, HJB16788). Municipality of Nanacamilpa, 19.4925°N, 98.5778°W, alt. approx. 2725 m, on burnt soil and litter in coniferous woodland under *Pinusmontezumae*, 6 Jul 2017, Forayer (TLXM HJB16747). Municipality of Nanacamilpa, 19.4923°N, 98.5783°W, alt. approx. 2730 m, on burnt soil and litter in coniferous woodland under *Pinusmontezumae*, 6 Jul 2017, A. Kong (TLXM HJB16748). Municipality of Nanacamilpa, road from Nanacamilpa to Tepuente, 19.4922°N, 98.5783°W, alt. approx. 2730 m, on burnt soil and litter in coniferous woodland under *Pinusmontezumae*, 6 Jul 2017, A. Montoya-Esquivel (TLXM HJB16749). Municipality of Nanacamilpa, road from Nanacamilpa to Tepuente, 19.4928°N, 98.5792°W, alt. approx. 2725 m, on burnt soil and litter in coniferous woodland under *Pinusmontezumae*, 6 Jul 2017, L. Davies (TLXM HJB16750). Municipality of Nanacamilpa, 19.4928°N, 98.5792°W, alt. approx. 2725 m, on burnt soil and litter in coniferous woodland under *Pinusmontezumae*, 6 Jul 2017, L. Davies (TLXM HJB16751). Municipality of Nanacamilpa, 19.4933°N, 98.5791°W, alt. approx. 2725 m, on burnt soil and litter in coniferous woodland under *Pinusmontezumae*, 6 Jul 2017, L. Davies (TLXM HJB16752). Municipality of Nanacamilpa, 19.4935°N, 98.579°W, alt. approx. 2725 m, on burnt soil and litter in coniferous woodland under *Pinusmontezumae*, 6 Jul 2017, L. Davies (TLXM HJB16753). Municipality of Panotla, San Mateo, Huexoyucan, 19.3874°N, 98.3028°W, alt. approx. 2485 m, on soil in deciduous woodland under *Quercus* sp., 10 Jul 2017, H.J. Beker (TLXM HJB16820). Municipality of Santa Ana Chiahutempan, La Malinche National Park, Surroundings of San Pedro Tlalcuapan, approx. 19.2152°N, 97.9841°W, alt. approx. 3100 m, on soil, 18 Jul 1998, A. Montoya-Esquivel (TLXM AME1652, HJB16766). Municipality of Tlaxco, north of El Rosario, El Rodeo, approx. 19.3395°N, 99.3605°W, alt. approx. 3100 m, on soil in woodland under *Pinus* sp. and *Quercus* sp., Jun 1991, A. Kong (TLXM AK1925, HJB16787). Municipality of Tlaxco, north of El Rosario, El Rodeo, approx. 19.2153°N, 97.9841°W, alt. approx. 3100 m, on soil in woodland under *Pinus* sp. and *Quercus* sp., 10 Jul 1991, A. Kong (TLXM AK1972, HJB16790). Municipality of Trinidad Sánchez Santos, La Malinche National Park, east of Javier Mina, approx. 19.2152°N, 97.9841°W, alt. approx. 3100 m, on soil, 21 May 1994, Hernandez-Valencia (TLXM HV6, HJB16778). Municipality of Trinidad Sánchez Santos, La Malinche National Park, east of Javier Mina, approx. 19.2153°N, 97.9841°W, alt. approx. 3100 m, on soil in woodland under *Alnus* sp. and *Pinus* sp., 3 Jul 1998, A. Montoya-Esquivel (TLXM AME1643, HJB16781). Tlaxcala City, mushrooms bought at the Tlaxcala market, 10 Jul 1999, A. Montoya-Esquivel (TLXM AME1713, HJB16764). Tlaxcala City, bought in market at Tlaxcala, collected from La Malinche National Park, 19.3218°N, 98.2387°W, alt. approx. 2160 m, 8 Jul 2017, M.F.M. Aguilar (TLXM HJB16809). Tlaxcala City, bought in market at Tlaxcala, collected from La Malinche National Park, 19.3218°N, 98.2388°W, alt. approx. 2160 m, 8 Jul 2017, M.F.M. Aguilar (TLXM HJB16810).

##### Remarks.

With its small ellipsoid, non-dextrinoid basidiospores and cheilocystidia swollen in the lower half, often lageniform or ventricose, this taxon clearly belongs to Hebelomasect.Hebeloma and is closely related to the complex of species around *H.mesophaeum*. The close, but not crowded, lamellae with more than 50 full length lamellae rules out *H.excedens* and *H.mesophaeum*, both of which are widespread throughout North America (Eberhardt et al. 2022a). Indeed, were this mushroom collected in Europe, and the key of Beker et al. (2016) applied, this would key out to *H.subtortum*. *Hebelomasubtortum* is most common in southern Europe, growing with lowland pines, and not known from North America. Within North America, no known taxon in H.sect.Hebeloma with such small ellipsoid spores has this number of full-length lamellae, making these characters sufficient for its determination.

**Figure 6.**
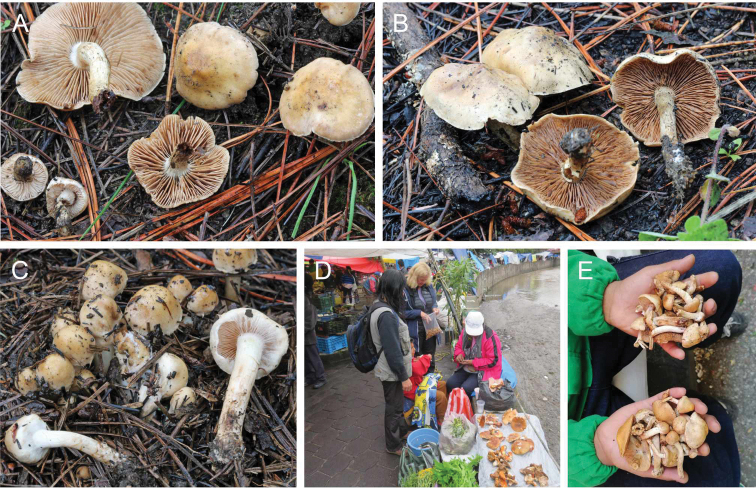
*Hebelomaambustiterranum***A–C** basidiomata **A** holotype TLXM 6155 (HJB16802) **B**TLXM HJB16803. **C**TLXM HJB16805 **D** mushroom vendor in the market of Tlaxcala City **E***H.ambustiterranum* sold in the market of Tlaxcala City. Photos **A–D**H.J. Beker **E** A. Montoya.

Fig. 6D–E show this mushroom for sale in local markets of Tlaxcala, where it is regarded as a prized edible mushroom known as hongo de ocote (ocote mushroom) in Spanish (Montoya et al. 2002). It is gathered from the temperate pine woodlands at altitudes of 2000 m and above. The local people burn the ground in the pine forests, ahead of the growing season, to encourage the growth of this mushroom. Frequent, controlled fires prevent the development of hot fires that would also damage the pines and pine roots, which are required for the fungi to grow. It is referred to in Nahuatl by several names, for example as the Xolete de ocō-xāl or ocō-xāl-nanácatl (ocō-xālli = pine-litter; mushroom growing in ocō-xāl - the mushroom of the pine needles), rastrojo-nanácatl (mushroom growing on stubble), ocochalero, ocotero, ocoxal, ocochal, cholete de ocote, nixtamalero or as chamusquinero, meaning from burnt ground (Estrada-Martínez et al. 2009; Reyes-López et al. 2020; Viveros-Assad et al. 2019). It is likely the same species as mentioned by Guzmán (1977) as “joletes” in Spanish, described as commonly sold in the Amecameca market, where it is recommended to boil them and then discard the water so that they are safe for consumption.

**Figure 7.**
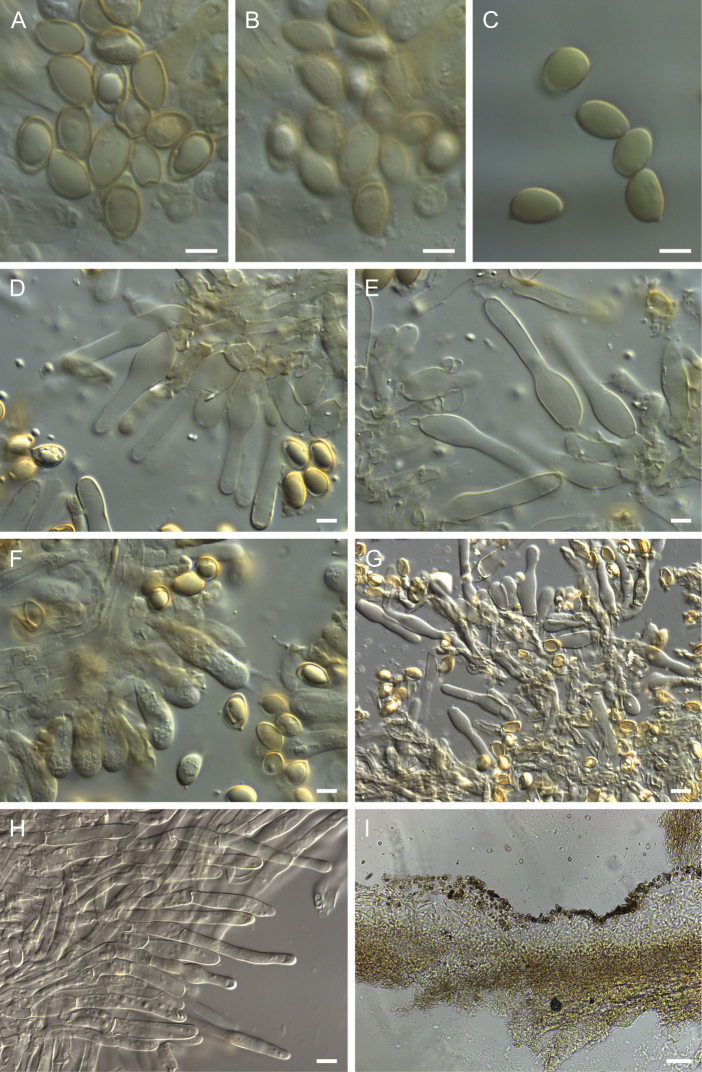
Holotype of *Hebelomaambustiterranum*TLXM 6155 (HJB16802) **A** basidiospores ×1600 **B** spore ornamentation ×1600 **C** basidiospores in Melzer’s reagent ×1600 **D–E** cheilocystidia ×1000 **F** basidia ×1000 **G** cheilocystidia on lamella edge ×500 **H** caulocystidia ×500 **I** Cutis ×125. All in KOH, except **C**. Scale bars: 5 µm (**A–F**); 10 µm (**G–J**); 50 µm (**K**). Photos H.J. Beker.

#### ﻿ Hebeloma cohaerens

Taxon classificationFungiAgaricalesHymenogastraceae

A. Montoya & Beker, sp. nov.

CBDB0995-27EE-5EA4-82CE-1722CFFC9B82

842828

Figs 8 , 9

##### Type.

Mexico. Tlaxcala: Municipality of Panotla, 1 km al este de San Francisco Temezontla, approx. 19.3496°N, 98.2784°W, alt. approx. 2600 m, in deciduous woodland under *Quercus* sp., 23 Jul 2017, A. Montoya-Esquivel AME3102 (holotype TLXM 6156; isotype BR 5020224875654V; HJB17733); Genbank ITS ON202511.

##### Diagnosis.

The short clavate-ventricose cheilocystidia, with average apical width less 6.5 µm, the small (on average less than 10 × 5.5 µm), weakly ornamented but rather strongly dextrinoid basidiospores and the whitish to cream or buff color of the pileus, differentiate this species from other *Hebeloma* species.

##### Etymology.

From *cohaerens* (adj. Latin) meaning united or joined together, to emphasize the connate habitus.

##### Description.

Pileus (22) 32–38 (47) mm diameter, convex, often applanate, occasionally umbonate; margin smooth, often involute, particularly when young, occasionally eroded, not hygrophanous; usually almost unicolored, usually cream or buff, sometimes slightly paler towards margin. Lamellae often adnate or adnexed, occasionally emarginate, depth up to 4 mm, white, cream to brown, with white fimbriate edge but without droplets on the lamella edge, number of full-length lamellae 70–80. Stipe (31) 37–46 (48) mm long, (5) 7–8 (10) mm diameter at median, usually cylindrical but sometimes with a clavate base, surface cream, ivory, not discoloring, fibrillose, pruinose, particularly towards apex; base with white mycelium. Context in pileus and stipe white to cream, firm, in stipe stuffed; taste not recorded, smell earthy. Spore deposit color not recorded.

Basidiospores based on n = 64 spores of the holotype, 5% to 95% percentile range 8.6–10.5 × 4.9–5.7 µm, with median 9.4 × 5.3 µm and av. 9.5 × 5.3 µm with S.D. length 0.57 µm and width 0.26 µm; Q value 5% to 95% percentile range 1.64–1.95, with median 1.79 and av. 1.78 with S.D. 0.10; spore size based on four collections medians 9.1–9.5 × 5.3–5.6 µm and av. 9.1–9.5 × 5.3–5.5 µm with av. S.D. length 0.50 µm and width 0.30 µm, av. Q 1.65–1.78, amygdaloid, occasionally limoniform, with small apiculus and rounded apically, often subacute, with a distinct thinning of the apical wall and sometimes a papilla, usually guttulate with one or sometimes more oily drops, at most weakly ornamented (ornamentation only visible under immersion), with a perispore hardly loosening, rather strongly dextrinoid, becoming medium reddish brown in Melzer’s reagent (O1/2; P0; D3); yellow brown in KOH. Basidia 22–27 × 5–7 µm, with av. Q 3.7–3.8 µm, cylindrical to clavate, hyaline, 4–spored. Cheilocystidium width near apex holotype 5% to 95% percentile range 4.7–7.7 µm, with median 6.0 µm and av. 6.1 µm with S.D. 1.0 µm; across four collections median 5.6–6.4 µm and av. 5.5–6.3 µm; examining approx. 20 selected cheilocystidia of each of the four collections yields a range for the avs. of 33–36 × 5.5–6.3 × 3.5–4.1 × 5.5–6.6 µm and 33 × 6.1 × 4.1 × 6.5 µm av. for holotype. Cheilocystidium av. ratios A/M: 1.49–1.63, A/B: 0.86–1.03, B/M: 1.59–1.88, mainly clavate-ventricose, often with one or two septa. Pleurocystidia absent. Caulocystidia similar to cheilocystidia but larger, up to 90 μm long. Pileipellis an ixocutis with an epicutis up to 110 µm thick, with gelatinized, hyphae up to 6 µm wide; subcutis cream to pale yellow, and the trama below the cutis made up of cylindrical, often ellipsoid cells, up to 14 µm wide. Clamp connections present throughout the basidiome.

##### Ecology and distribution.

In deciduous or mixed woodlands apparently associated with *Quercus* or *Pseudotsuga*. Growth habit mainly caespitose, sometimes with a few scattered basidiomes. To date, all collections of *Hebelomacohaerens* recorded from Tlaxcala at altitudes of 2600 m or more.

##### Additional collections examined.

Mexico. **Tlaxcala**: Municipality of Panotla, 1 km al este de San Francisco Temezontla, approx. 19.3496°N, 98.2784°W, alt. approx. 2600 m, in deciduous woodland under *Quercus* sp., 23 Jul 2017, A. Montoya-Esquivel (TLXM AME3101, HJB17732). Municipality of Panotla, 1 km al este de San Francisco Temezontla, approx. 19.3496°N, 98.2784°W, alt. approx. 2600 m, in deciduous woodland under *Quercus* sp., 23 Jul 2017, A. Kong (TLXM AK17-08, HJB17737). Municipality of Terrenate, Rancho el pozo, approx. 19.5407°N, 97.9046°W, alt. approx. 2900 m, on soil in woodland under *Pseudotsuga* sp., 13 Jul 1995, Galindo-Flores (TLXM GF1866, HJB16779).

##### Remarks.

The small, short clavate-ventricose cheilocystidia, together with the small rather smooth, rather strongly dextrinoid basidiospores, support the placement of this species within Hebelomasect.Theobromina. Within this section the pale cream to buff pileus color together with the caespitose habitus is unique.

The description was based on just four collections all from the same region of Mexico, and is not known from any other location. More records for this species will help to define better its morphological characters and its biogeographic preferences.

The minimum interspecific distance between the ITS sequences of *H.cohaerens* and sequences from other species is around 1.2%. The BLAST result of the type sequence of *H.cohaerens* against UNITE resulted in a hit of a soil sample sequence, pointing towards UNITE SH1563973.08FU (98.5% level). This SH includes two independently generated sequences from California (UDB0767851, soil sample, Tedersoo et al. 2021; DQ822802, basidiome, Point Reyes National Seashore Reserve, under *Pinusmuricata*, Peay et al. 2007) that differ by around 0.5% from the sequences assigned to *H.cohaerens*, but match no other species. These results suggest that *H.cohaerens* may occur in the US (California) and the UNITE SH corresponding to *H.cohaerens* is likely to be SH1563973.08FU.

**Figure 8.**
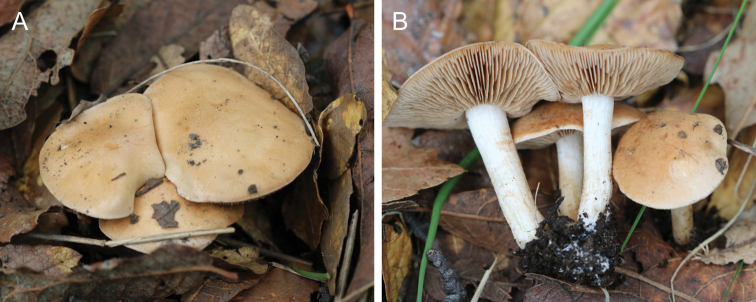
**A–B** basidiomata, holotype of *Hebelomacohaerens*TLXM 6156 (HJB17733). Photos A. Kong.

**Figure 9.**
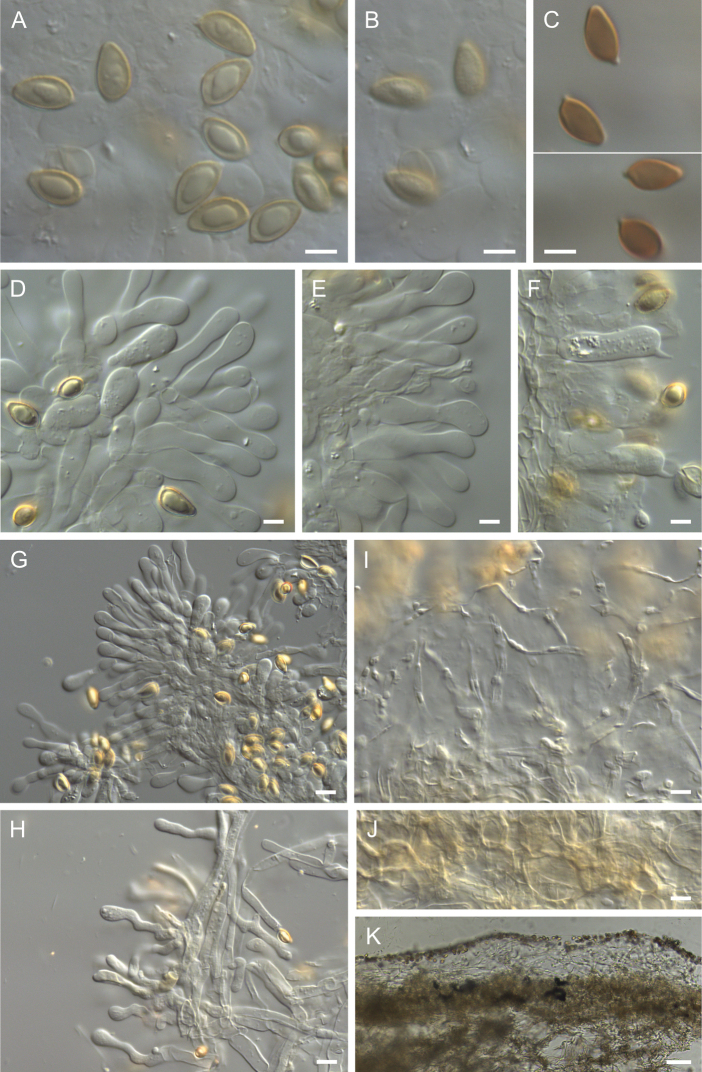
Holotype of *Hebelomacohaerens*TLXM 6156 (HJB17733) **A** basidiospores ×1600− **B** spore ornamentation ×1600 **C** basidiospores in Melzer’s reagent ×1600 **D–E** cheilocystidia ×1000 **F** basidia ×1000 **G** cheilocystidia ×500 **H** caulocystidia ×500 **I** epicutis hyphae ×1000 **J** subcutis ×1000 **K** cutis ×125. All in KOH, except **C**. Scale bars: 5 µm (**A–F**); 10 µm (**G–J**); 50 µm (**K**). Photos H.J. Beker.

#### ﻿ Hebeloma magnicystidiatum

Taxon classificationFungiAgaricalesHymenogastraceae

A. Kong & Beker, sp. nov.

A55085BB-0557-55BD-95D5-7683F1957E69

842829

Fig. 10

##### Type.

Mexico. Tlaxcala: Municipality of Totolac, Tepeticpac, 19.3457°N, 98.2226°W, alt. approx. 2400 m, on the ground in woodland under *Pinus* sp. and *Quercus* sp., 29 Aug. 1990, A. Estrada-Torres AET3093 (holotype TLXM 6157; isotype BR 5020224873599V; HJB16795); GenBank ITS ON202534.

##### Diagnosis.

The amygdaloid, non-dextrinoid, rather strongly ornamented spores with average Q value less than 1.6 and the capitate-stipitate cheilocystidia with average width at the apex greater than 9.5 µm distinguish this species from all other known *Hebeloma* species.

##### Etymology.

From *magni*- (Latin, composite) meaning large and *cystidiatus* to emphasize the large capitate-stipitate cheilocystidia.

##### Description.

Pileus 19–26 mm diameter, convex, surface dry, finely tomentose, cuticle separable, reddish yellow to brown in the center, and pale orange towards the margin. Lamellae emarginate, white, cream to orange brown as the spores mature, with a white fimbriate edge, and about 60 full-length lamellae. Stipe 10–21 mm long, 4–6 mm diameter at median, cylindrical, surface whitish but discoloring brown from the base upwards, with age or handling, fibrillose, at apex pruinose. Context in pileus white to cream, firm, in stipe stuffed, initially white to cream but becoming brown with age and handling, becoming hollow with age; taste fungal to sweet, smell raphanoid. Spore deposit not recorded.

Basidiospores based on n = 44 spores of the holotype, 5% to 95% percentile range 9.7–11.6 × 6.4–7.6 µm, with median 10.5 × 7.0 µm and av. 10.5 × 7.0 µm with S.D. length 0.62 µm and width 0.42 µm; Q value 5% to 95% percentile range 1.40–1.62, with median 1.49 and av. 1.50 with S.D. 0.07; amygdaloid, often limoniform, with small apiculus and rounded apically, often subacute to acute, with a distinct thinning of the apical wall and sometimes a clearly visible papilla, not guttulate, usually rather strongly ornamented, ornamentation visible even without immersion, with perispore at most somewhat loosening in a few spores, an indistinct brownish tint in Melzer’s reagent (O3; P1; D1); yellow-brown in KOH. Basidia 27.5–35 × 7.5–9 µm, with av. Q 3.9, cylindrical to clavate, without pigmentation, 4-spored. Cheilocystidium width near apex holotype 5% to 95% percentile range 6.1–14.3 µm, with median 9.1 µm and av. 9.7 µm with S.D. 2.61 µm; examining approx. 20 selected cheilocystidia of the holotype yields a range for the avs. of 55 × 9.7 × 7.3 × 4.3 µm av. and cheilocystidium av. ratios A/M: 2.58, A/B: 2.67, B/M: 0.95; mainly capitate-stipitate, unfortunately many collapsed in exsiccata. Pleurocystidia absent. Caulocystidia similar to cheilocystidia but larger, up to 80 μm long. Pileipellis an ixocutis; epicutis up to 110 µm thick, with gelatinized hyphae up to 7 µm wide; subcutis yellow; and the trama below the cutis made up of cylindrical or occasionally ellipsoid cells up to 17 µm wide. Clamp connections present throughout the basidiome.

##### Ecology and distribution.

In woodland on the ground with *Comarostaphylis* and *Quercus*. Growth habit scattered. To date, with only one collection of this species, not possible to describe its distribution and ecology.

##### Remarks.

With its amygdaloid, hardly dextrinoid basidiospores and capitate-stipitate cheilocystidia, morphologically this taxon clearly belongs to Hebelomasect.Denudata and there to H.subsect.Crustuliniformia. The amygdaloid spores with rather small average Q value separate this species from all other studied *Hebeloma* from our database with more than 10,000 collections. While this may suggest that this is a rare species, we have insufficient *Hebeloma* collections from Mexico to reach such a conclusion. The single collection was collected in the 1990s, thus the only loci we could amplify were ITS and mitSSU variable regions V6 and V9.

The phylogenetic placement of *H.magnicystidiatum* within H.sect.Denudata is unresolved. As pointed out before (e.g. Eberhardt et al. 2016, 2022b; Beker et al. 2016), the more species rich subsections of H.sect.Denudata (*H.* subsects *Clepsydroida* and *Crustuliniformia*) are not supported molecularly. In terms of ITS, the most similar species was *H.sordidulum* (H.subsect.Clepsydroida) with similarity values ≤ 98.7%. Possibly *H.magnicystidiatum* will correspond to a UNITE SH at the 99% or 98.5% level once sequences of this species are included in the system. Morphologically, the capitate-stipitate cheilocystidia together with the amygdaloid spores with av. Q less than 1.6 are sufficient characters to separate this species from members of H.sect.Clepsydroida, such as *H.cavipes*, *H.matritense*, *H.sordidulum* and *H.vaccinum*.

**Figure 10.**
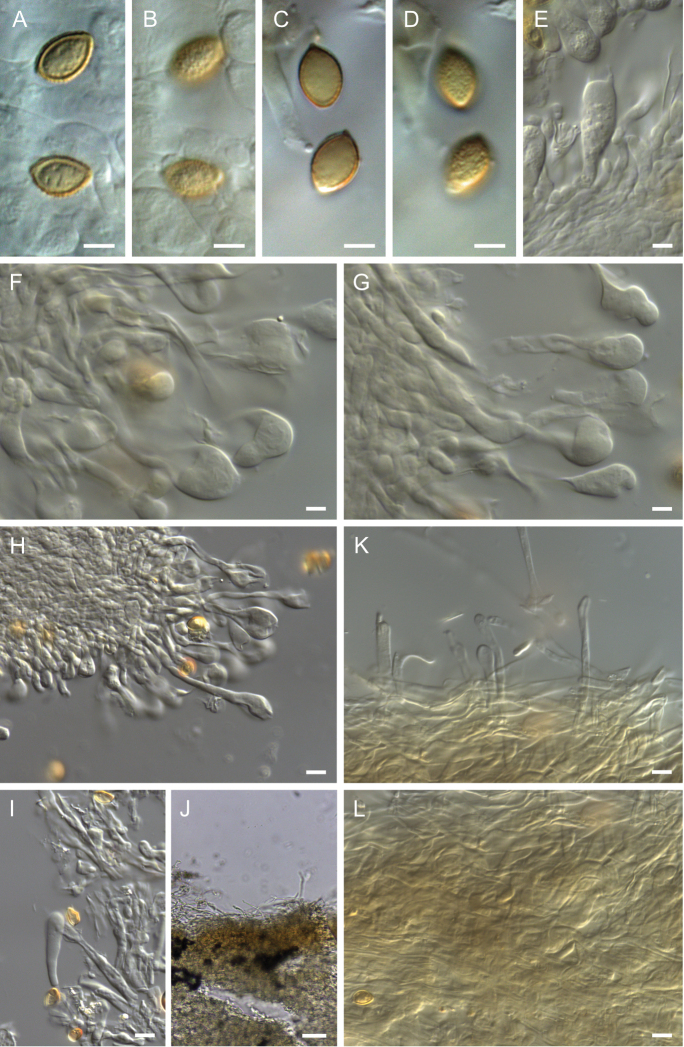
Holotype of *Hebelomamagnicystidiatum*TLXM 6157 (HJB16795) **A** Basidiospores ×1600 **B** spore ornamentation ×1600 **C** basidiospores and **D** spore ornamentation in Melzer’s reagent ×1600 **E** basidium ×1000 **F–H** cheilocystidia ×1000 **H** cheilocystidia ×500 **I** caulocystidia ×500 **J** cutis ×125 **K** epicutis hyphae ×500 **L** subcutis ×500. All in KOH, except **C–D**. Scale bars: 5 µm (**A–G**); 10 µm (**H–I, K–L**); 50 µm (**J**). Photos H.J. Beker.

#### ﻿ Hebeloma neurophyllum

Taxon classificationFungiAgaricalesHymenogastraceae

G.F. Atk., Annales Mycologici 7(4): 370 (1909)

F82EBAA4-645C-5A0B-877F-AE8927D1C4ED

Figs 11 , 12

##### Type.

USA. New York: Coy Glen, Ithaca, approx. 42.4272°N, 76.5241°W, alt. approx. 125 m, on soil in woodland, 18 Oct 1906, N. Coil (holotype CUP-A-021514; isotype TENN-F-037531, HJB1000453, isotype WTU-F-039596, HJB1000558).

##### Diagnosis.

Gregarium 7–8 cm altum, pileo 5–6 cm lato, stipite 5–6 mm crasso: Pileo ochraceo-cremeo vel fulvo-ochraceo, leviter viscido. Lamellis 8 mm latis, pallide cinnamomeo-rufis, late sinuatis, adnexis, costatis. Basidiis 4-sporis. Sporis subfusoideis, 12–15 × 7–8 µ[m]. Ad terram in silvis, Ithacae, N. Y. Stipite albo, fibroso-striato, cavo vel subfarcto.

##### English translation of diagnosis.

Gregarious 7–8 cm high, pileus 5–6 cm broad, stipe 5–6 mm thick: pileus ochraceous-cream or fulvous-ochraceous, slightly viscid. Lamellae 8 mm broad, pale cinnamon-reddish, broadly sinuate, adnexed, intervenose. Basidia four-spored. Spores subfusoid, 12–15 × 7–8 μm. On the ground in woodland, New York. Stipe white, fibrous-striate, fistulose or almost stuffed.

##### Description.

Pileus (26) 30–55 (60) mm diameter, convex, occasionally umbonate or broadly umbonate; margin often smooth, occasionally involute or wavy, not hygrophanous; usually unicolor, occasionally two colors, at center occasionally yellowish brown, ochraceous or cream, rarely fawn, cinnamon or clay-buff, sometimes slightly paler towards margin. Lamellae usually emarginate, occasionally adnexed, depth up to 9 mm, white, cream to brown, usually with white fimbriate edge, usually without droplets on the lamella edge but rarely some drops may be visible, number of full-length lamellae 70–94. Stipe (25) 31–75 (80) mm long, 5–14 (16) mm diameter at median, often clavate or bulbous, occasionally cylindrical, (7) 9–16 (18) mm wide at base, surface cream, ivory, rarely discoloring, occasionally velutinous, floccose or fibrillose, often pruinose, particularly towards apex. Veil not observed. Context in pileus white to cream, firm, in stipe usually hollow, rarely with superior hanging wick; taste mild, smell occasionally raphanoid or odorless, rarely fruity or earthy. Spore deposit yellowish brown to brownish olive.

Basidiospores based on n = 70 spores of the holotype, 5% to 95% percentile range 12.7–15.6 × 7.2–9.0 µm, with median 14.2 × 8.2 µm and av. 14.2 × 8.2 µm with S.D. length 0.93 µm and width 0.54 µm; Q value 5% to 95% percentile range 1.52–1.91, with median 1.74 and av. 1.73 with S.D. 0.12; spore size based on 47 collections medians 11.6–14.3 × 7.2–8.2 µm and av. 11.7–14.2 × 7.5–8.3 µm with av. S.D. length 0.898 µm and width 0.459 µm, av. Q 1.53–1.78, amygdaloid, usually limoniform, with small apiculus and rounded apically, often subacute to acute, with a distinct thinning of the apical wall and a clear papilla, occasionally guttulate with one or sometimes more oily drops, distinctly to strongly ornamented (ornamentation visible without immersion), with a perispore somewhat to distinctly loosening, at least in a few spores, strongly dextrinoid, becoming at least medium brown and often intensely red-brown in Melzer’s reagent (O3/4; P1/2; D3/4); yellow to brown in KOH. Basidia 20–43 × 7–10 µm, with av. Q 2.7–3.8 µm, cylindrical to clavate, with a median constriction, hyaline, 4-spored. Cheilocystidium width near apex holotype 5% to 95% percentile range 4.9–9.0 µm, with median 6.5 µm and av. 6.7 µm with S.D. 1.27 µm; across 47 collections median 4.5–6.8 µm and av. 4.6–6.7 µm; examining approx. 20 selected cheilocystidia of each of the 47 collections yields a range for the avs of 40–59 × 4.6–6.7 × 4.4–5.7 × 5.6–8.4 µm and 49 × 6.7 × 5.6 × 6.7 µm av. for the holotype. Cheilocystidium av. ratios A/M: 1.01–1.41, A/B: 0.68–1.23, B/M: 1.16–1.58, mainly gently clavate or ventricose, occasionally cylindrical, lageniform or clavate-lageniform or clavate-ventricose, often with one or two septa, sometimes clamped, often with plaques on the cystidial walls, occasionally geniculate or with basal wall thickening, rarely bifurcate, hyaline, rarely with yellow contents. Pleurocystidia absent. Caulocystidia similar to cheilocystidia but larger, up to 115 μm long. Pileipellis an ixocutis, epicutis up to 90 µm thick, with gelatinized, hyphae up to 6 µm wide; subcutis pale yellow to brownish yellow, and the trama below the cutis made up of cylindrical, often ellipsoid cells, up to 16 µm wide. Clamp connections present throughout the basidiome.

##### Habitat and distribution.

Based on almost 50 collections, where only one possible associate was recorded, the most commonly recorded associates were *Picea* and *Quercus*, but *Populus*, *Salix* and *Tilia* were also recorded; the most commonly recorded families were Fagaceae, Pinaceae and Salicaceae, but Betulaceae and Malvaceae were also recorded. We have additional records where *Alnus*, *Arctostaphylos*, *Betula*, *Dryas*, *Pinus* and *Polygonum* were recorded as possible associates, but in each of these cases a number of possible associates were mentioned. All records of *H.neurophyllum* are from Northern America, where it is widespread across the region but primarily collected in temperate to boreal woodland, occasionally in urban areas.

##### Additional material examined.

Canada. **Alberta**: Moose Hill, Breton, Edmonton, 53.1418°N, 114.6097°W, alt. approx. 810 m, on soil in mixed woodland under *Piceamariana*, 12 Aug 2017, H.J. Beker (HJB16856). **Northwest Territories**: Highway 3, between Yellowknife and Behchoko, 62.5198°N, 114.897°W, alt. approx. 165 m, on mossy soil in boreal, calcareous woodland roadside under *Betula* sp. and *Salix* sp., 7 Sep 2018, H.J. Beker, L. Davies (HJB18101). **Yukon**: Railway Station, Whitehorse, 60.7214°N, 135.0505°W, alt. approx. 665 m, on soil and litter in boreal shrubland riverside under *Populustremuloides* and *Salix* sp., 31 Aug 2018, H.J. Beker, L. Davies (HJB17975). 3^rd^ Avenue near Wood St intersection, Whitehorse, 60.7212°N, 135.0555°W, alt. approx. 665 m, on grassy, mossy soil in boreal urban roadside under *Populus* sp., 1 Sep 2018, H.J. Beker, L. Davies (HJB17981). MEXICO. **Chihuahua**: El Ranchito, approx. 28.3387°N, 105.4076°W, alt. approx. 1150 m, on soil in montane, subtropical woodland, 18 Aug 2001, A. Kong 3782 (TLXM AK3782, HJB16773). UNITED STATES. **Alaska**: Kantishna Roadhouse Nature Trail, Denali National Park, 63.5243°N, 150.9625°W, alt. approx. 490 m, on sandy soil in boreal, mixed but mainly coniferous woodland pathside under *Alnus* sp., *Betula* sp. and *Salix* sp., 18 Aug 2018, H.J. Beker, L. Davies (DENA-61424, HJB17897). **Texas**: Jefferson County, Beaumont, residence of Penny Clark, approx. 30.0788°N, 94.1372°W, alt. approx. 0 m, in garden under *Quercusfusiformis*, 4 Dec 2015, D. Lewis DPL11907 (HJB15699). **Wisconsin**: Bark Point Road, near Bark Bay, 46.8353°N, 91.2594°W, alt. approx. 185 m, on grassy soil in coniferous garden under *Piceaglauca*, 13 Sep 2017, L. Davies, H.J. Beker (HJB16991).

##### Remarks.

With the mixture of gently clavate and ventricose cystidia alongside the strongly dextrinoid basidiospores, this species belongs within Hebelomasect.Velutipes. Within this section the combination of spores with minimum average width 7.5 µm and a distinctly loosening perispore in at least some spores, together with the absence of pleurocystidia, defines this species. The collection of *H.neurophyllum* from Mexico, gathered at El Ranchito in Chihuahua, matches well with other collections of this species. We are not aware of any synonyms for this species.

In terms of ITS, the most similar to *H.neurophyllum* were *H.celatum*, *H.erebium* and *H.quercetorum*, the ITS sequences of which were around 99% similar (99.2–98.6%) to those of *H.neurophyllum*. *Hebelomaneurophyllum* appears to correspond to UNITE SH1733487.08FU (99%). Intriguingly, this species hypothesis includes a number of soil sample sequences from Estonia, suggesting that either *H.neurophyllum* occurs in Europe, too, or that species known to occur in Europe also contain ITS copies corresponding to *H.neurophyllum* below the detection limit of Sanger sequencing.

**Figure 11.**
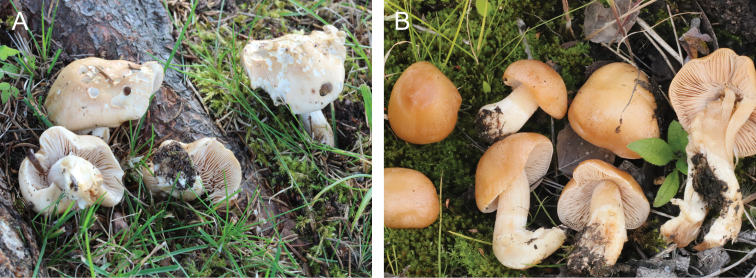
*Hebelomaneurophyllum*, basidiomata **A** HJB16991 **B** HJB18101. Photos H.J. Beker.

**Figure 12.**
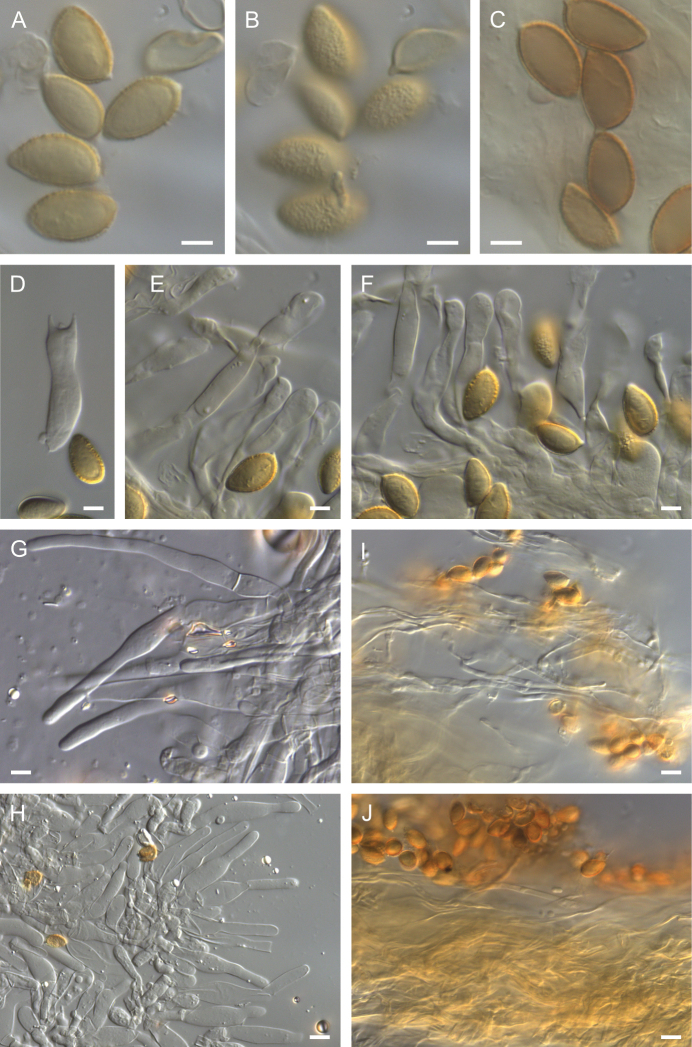
*Hebelomaneurophyllum***A** basidiospores and **B** spore ornamentation of isotype TENN-F-037531 (HJB1000453) ×1600 **C** basidiospores of HJB17897 in Melzer’s reagent ×1600 **D** basidium of isotype ×1000 **E–F** cheilocystidia of isotype ×1000 **G** caulocystidia of HJB17975 ×500 **H** caulocystidia of HJB16856 ×500 **I** epicutis hyphae and **J** subcutis of isotype ×500. All in KOH, except **C**. Scale bars: 5 µm (**A–F**); 10 µm (**G–J**). Photos H.J. Beker.

#### ﻿ Hebeloma subaustrale

Taxon classificationFungiAgaricalesHymenogastraceae

Murrill, Lloydia 8: 287 (1946) [1945]

36429271-02CF-5230-8080-9C2E8D57D8FF

Fig. 13

= Hebelomaangustisporium Hesler, Kew Bulletin 32(3): 471 (1977)
= Hebelomaperangustisporium Hesler, Kew Bulletin 32(3): 478 (1977)

##### Type.

USA. Florida: Gainesville, Alachua Co., approx. 29.651634°N, 82.324826°W, alt. approx. 50 m, on grassy, shady soil in lawn, 30 Oct 1941, G.F. Weber (holotype FLAS-F-19345, HJB1000402; isotype TENN-F-021177, HJB1000447).

##### Diagnosis.

Pileo convexo-expanso, 3–4 cm. lato, subviscido, glabro, pallido-roseo, raphanico; lamellis sinuatis, latis, confertis; sporis subovoidcis, pallidis, levibus, 8–10 × 4–4.5 μ[m]; stipite acquali, pallido, 3 × 0.5 cm.

##### English translation of diagnosis.

Pileus convex to applanate, 3–4 cm broad, slightly viscid, glabrous, pale pink, with raphanoid smell; lamellae sinuate, broad, crowded; spores subovoid, pale, smooth, 8–10 × 4–4.5 μ[m]; stipe equal, pale, 3 × 0.5 cm.

##### Description.

Pileus (20) 32–45 (46) mm diameter, usually convex, occasionally umbonate; occasionally with remains of universal veil; margin often smooth, occasionally scalloped, not hygrophanous; usually unicolor, occasionally two colors, at center cream to buff to ochraceous, often becoming paler towards the margin. Lamellae usually emarginate, occasionally adnate or adnexed; white, cream to brown, usually with white fimbriate edge, without droplets on the lamella edge, number of full-length lamellae 80–92. Stipe 30–56 (70) mm long, 5–10 (11) mm diameter at median, often clavate or cylindrical, 5–13 (14) mm wide at base, surface cream, ivory to white rarely discoloring, pruinose, particularly towards apex. Context in pileus white to cream, firm, similar color in stipe, becoming hollow with age; taste raphanoid, smell raphanoid, occasionally earthy. Spore deposit cinnamon color.

Basidiospores based on n = 63 spores of the holotype, 5% to 95% percentile range 8.4–9.8 × 4.6–5.2 µm, with median 9.0 × 4.8 µm and av. 9.0 × 4.9 µm with S.D. length 0.51 µm and width 0.18 µm; Q value 5% to 95% percentile range 1.65–2.03, with median 1.88 and av. 1.85 with S.D. 0.12; spore size based on seven collections medians 8.5–10.2 × 4.6–5.3 µm and av. 8.6–9.9 × 4.6–5.3 µm with av. S.D. length 0.657 µm and width 0.271 µm, av. Q 1.73–2.09, amygdaloid, usually fusoid, rarely navicular, with small apiculus and rounded apically, often subacute to acute, with a distinct thinning of the apical wall and no papilla, occasionally guttulate with one or sometimes more oily drops, very weakly ornamented (ornamentation only visible under immersion), with a perispore somewhat loosening, in at most a few spores, rarely not loosening or distinctly loosening, distinctly to rather strongly dextrinoid, becoming yellow brown to medium brown in Melzer’s reagent (O1/2; P0/1/2; D2/3); yellow in KOH. Basidia 19–32 × 5–7 µm, with av. Q 3.8–4.6 µm, cylindrical to clavate, hyaline, 4-spored. Cheilocystidium width near apex holotype 5% to 95% percentile range 4.5–6.8 µm, with median 5.8 µm and av. 5.7 µm with S.D. 0.85 µm; across seven collections median 4.4–6.3 µm and av. 4.5–6.3 µm; examining approx. 20 selected cheilocystidia of each of the seven collections yields a range for the avs of 29–43 × 4.5–6.3 × 3.9–5.1 × 4.8–6.8 µm and 33 × 5.7 × 4.3 × 5.6 µm av. for the holotype. Cheilocystidium av. ratios A/M: 1.04–1.48, A/B: 0.84–1.31, B/M: 1.20–1.36, irregular but mainly cylindrical, often ventricose, often clavate, occasionally clavate-lageniform or clavate-ventricose or gently clavate, rarely capitate stipitate or clavate stipitate, often with one or two septa, occasionally with apical wall thickening. Pleurocystidia absent. Caulocystidia similar to cheilocystidia but larger, up to 100 μm. Pileipellis an ixocutis, epicutis up to 100 µm thick, with gelatinized, hyphae up to 7 µm wide, often encrusted; subcutis pale yellow; and the trama below the cutis made up of ellipsoid or thickly sausage-shaped, often cylindrical cells up to 13 µm wide. Clamp connections present throughout the basidiome.

##### Habitat and distribution.

Where only one possible associate was recorded, that associate has always been *Quercus* (Fagaceae). We have additional records where *Pinus*, *Abies* and *Fagus* were recorded as possible associates, but in each of these cases a number of possible associates were mentioned by the collector. We are only aware of five collections other than that from Mexico. These are all from the eastern half of the United States: Ohio, Pennsylvania and Tennessee.

##### Additional material examined.

Mexico. **Tlaxcala**: Municipality of Huamantla, La Malinche National Park, Cañada Grande, east side of La Malintzi volcano, approx. 19.1999°N, 97.9729°W, alt. approx. 3000 m, on soil in montane, temperate woodland under *Abies* sp. and *Pinus* sp., 25 Jul 1990, H. Cuevas HC1155 (TLXM HC1155, HJB16793). USA. **Ohio**: Shaker Parklands, Doan Brook Gorge, approx. 41.495°N, 81.5953°W, alt. approx. 275 m, on grassy soil under *Fagus* sp. and *Quercus* sp., 26 Sep 2011, D. Bartholow SPFS-2011-63 (HJB17796). **Pennsylvania**: Fort Washington Park, Parking Lot 5, approx. 40.1208°N, 75.2232°W, alt. approx. 80 m, on soil in mixed woodland under *Quercus* sp., 23 Oct 2018, T. Deluce (HJB18418). **Tennessee**: Gatlinburg, Great Smoky Mountains National Park, Indian Gap, approx. 35.6108°N, 83.4386°W, alt. approx. 1650 m, 29 Jul 1941, L.R. Hesler LRH13890 (holotype of *Hebelomaperangustisporium* TENN-F-013890, HJB1000450). Blount, Townsend, Great Smoky Mountains National Park, Cades Cove, approx. 35.6019°N, 83.8113°W, alt. approx. 550 m, 23 Aug 1959, L.R. Hesler LRH23364 (holotype of *Hebelomaangustisporium* TENN-F-023364, HJB1000314).

##### Remarks.

The small weakly ornamented basidiospores together with the short irregular cheilocystidia, often cylindrical but also both ventricose and clavate, suggest Hebelomasect.Naviculospora, which is supported by molecular data. Within this section *H.subaustrale* is differentiated from other Northern American species of this section by the average basidiospore length (a maximum of 10 µm), and average spore Q greater than 1.7, together with the cheilocystidia that have a maximum average A/B ratio of 1.5 and a minimum average B/M ratio of 1.2.

We were not able to generate any sequence data from the type of *H.subaustrale*. However, our morphological study of the type, and of a number of other species within H.sect.Naviculospora, leaves us in no doubt that this is a conspecific of both *H.angustisporium* and *H.perangustisporium*. For these latter two species types we have good morphological and molecular data. Table 1 shows a comparison of the most important taxonomic parameters for the holotypes of these three species. The spore size and the average cheilocystidium shape, despite their irregularity, are key to differentiating species within this section. The Mexican collection corresponded well with this type material and other recent collections from the USA.

**Table 1.** Comparison of the most taxonomically important holotype characters of *Hebelomasubaustrale* and its synonyms. Macroscopic data from the original descriptions and microscopic measures from own studies.

Species	*Hebelomaangustisporium*	*Hebelomaperangustisporium*	*Hebelomasubaustrale*
Number of complete lamellae	86	80	88
Spore ornamentation	O1; O2	O2	O1
Spore perispore loosening	P1	P1; P2	P0; P1
Spore dextrinoidity	D2; D3	D1; D2	D2
Spore length av. (µm)	8.6	9.9	9
Spore width av. (µm)	5	5.3	4.9
Spore Q av.	1.73	1.87	1.85
Cheilocystidia length av. (µm)	29	39	33
Cheilocystidia apex on gill edge av. (µm)	4.5	4.6	5.7
Cheilocystidia av. Q1, A/M	1.04	1.12	1.38
Cheilocystidia av. Q2, A/B	0.86	0.84	1.06
Cheilocystidia av. Q3, B/M	1.24	1.36	1.44
Basidia Q av.	4.3	3.8	3.8
Pileus diameter (mm)	25–40	20–45	30–40
Stipe median width (mm)	9–10	9–11	5

*Hebelomasubaustrale* formed a reasonably well supported clade in the ITS analysis (Fig. 5B), thus it is expected to be identifiable by its barcode. Although the maximum intraspecific distance of the sequences in the analysis is only 0.14%, the minimum distance to other species of the section is 0.7%. At this time (4 Feb 2022), there is no multi-sequence UNITE SH that represents the species; the published sequence of the holotype of *H.angustisporium* (NR_119890 = HQ179674) formed a singleton SH at the 99% level and the respective SH at the next level included several species.

**Figure 13.**
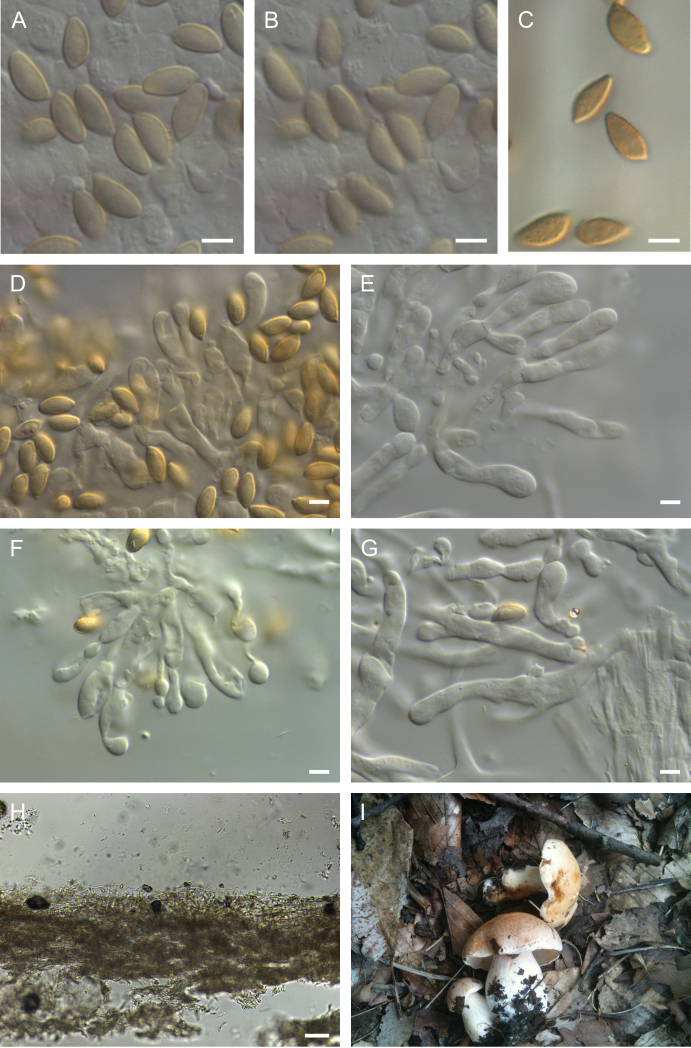
*Hebelomasubaustrale***A** basidiospores and **B** spore ornamentation of holotype FLAS-F-19345 ×1600 **C** spores of SPFS-2011-63 (HJB17796) in Melzer’s reagent ×1600 **D** spores and cheilocystidia of holotype ×1000 **E** cheilocystidia of isotype TENN-F-021177 ×1000 **F** cheilocystidia of holotype ×1000 **G** caulocystidia of isotype ×1000 **H** cutis of SPFS-2011-63 ×125. All in KOH, except **C**. **I** basidiomata of collection SPFS-2011-63. Scale bars: 5 µm (**A–G**); 50 µm (**H**). Photos **A–G**H.J. Beker **H** D. Bartholow.

## ﻿Discussion

The systematic position of the discussed species, three new (*H.ambustiterranum*, *H.cohaerens* and *H.magnicystidiatum*) and two neglected and rediscovered (*H.neurophyllum*, *H.subaustrale*), are unambiguous and supported by morphological and molecular results. All species can be placed in previously described sections of *Hebeloma*. Based on our current knowledge, all species are easy to delimit molecularly and are recognizable by their ITS-barcodes.

Garibay-Orijel et al. (2013) identified *H.albocolossum* (synonymized with *H.eburneum* by Beker et al. 2016), *H.helodes*, *H.leucosarx* and *H.mesophaeum* from ectomycorrhizal root tips of *Pinusmontezumae* from the Transmexican Volcanic Belt, based on the sequences available in GenBank at the time. The sequences of Garibay-Orijel et al. (2013) were not included in the tree analyses, because ITS only entries would have negatively influenced the phylogenetic resolution of the respective analyses. Based on currently available sequence data, we would tend to identify the sequences obtained in that study as *H.eburneum* (JN704820; species in Fig. 3), *H.excedens* or *H.mesophaeum* (JN704814; species in Fig. 2), *H.velutipes* (JN704825; species in Fig. 5), and *H.sordidulum* (JN704810; species in Fig. 3). These species are treated in detail by Beker et al. (2016) and Eberhardt et al. (2021a, 2022a). Given that these identifications are based only on ITS sequence data, they have to be treated with caution.

Many of the issues such as conflicting phylogenetic hypotheses or lack of species resolution in phylogenetic analyses have been encountered and discussed before for H.sect.Denudata (Eberhardt et al. 2015, 2016a; Beker et al. 2016), for H. sect. Velutipes (Aanen et al. 2001; Grilli et al. 2016; Beker et al. 2016) and H.sect.Hebeloma (Beker et al. 2016; Eberhardt et al. 2022a). For the delimitation and recognition of the species described in detail here, *H.ambustiterranum*, *H.cohaerens*, *H.magnicystidiatum*, *H.neurophyllum* and *H.subaustrale*, these are non-issues. For *H.magnicystidiatum* the conflicts between the different loci used imply that there was no molecular support for the assignment to subsection. However, already Eberhardt et al. (2016a) showed that even when using additional loci such as *RPB2*, *TEF1a* and *MCM7* support for *H.* subsects. *Clepsydroida* and *Crustuliniformia* was lacking and their relation to H.subsect.Hiemalia was unresolved. Likewise, Grilli et al. (2016) showed that the phylogenetic relationship between *H.celatum*, *H.erebium* and *H.quercetorum* could not be resolved based on five loci. Here, *H.neurophyllum* is presented as a fourth species in this group the evolutionary history of which could not be reconstructed based on four loci.

Other questions arising from the presented results will have to be tackled in a wider context with more samples, more loci and geographically wider sampling. These include whether *H.excedens* and *H.mesophaeum* should be treated as a single species (see also Eberhardt et al. 2022a), or whether to attach any importance to the somewhat isolated position of the Mexican *H.eburneum* in relation to other *H.eburneum* sequences in the analysis, or the divergent mitSSU V6 sequences of Mexican *H.velutipes*. Eberhardt and co-workers (2016) showed that member species of the *H.alpinum* complex varying in their mitSSU variable regions are likely to belong to different mating groups defined by Aanen and Kuyper (1999). Using the same reasoning, if the mitSSU V6 differences of the Mexican *H.eburneum* or *H.velutipes* had been accompanied by morphological differences, we would have had to recognize them as a distinct species. There were no differences found, thus the collections are here addressed as *H.eburneum* and *H.velutipes*, respectively, although the suspicion remains that the mitSSU results point towards mating groups—and possibly species—so far not sampled outside Mexico. Or, alternatively, that our current concept recognizes too many species in the respective groups.

There have been reports of edible *Hebeloma* species from other regions of the world, for example from Guatemala, Laos and Nigeria (Aremu et al. 2009; Carrasco-Hernández et al. 2015; Eberhardt et al. 2020a; Flores-Arzú 2020), where, for example, Eberhardt and colleagues reported that in Laos *H.parvisporum* is sold in markets and on roadsides as edible and that it is called “wai khom,” which refers to its bitter taste, which, apparently, remains, at least to some degree, after cooking.

From their literature review, Carrasco-Hernández et al. (2015) found that cytotoxic triterpenes, lanostanetype triterpene esters, neurotoxic cucurbitane-type glycosides and 6,7-seco-caryophyllenes, and related sesquiterpenoids may be the cause of *Hebeloma* toxicity. It is reported that *Hebeloma* poisonings typically cause gastrointestinal symptoms in humans that pass after several days. It is not known which species of *Hebeloma* are poisonous, but, as said above, their consumption is strongly discouraged (Bresinsky and Besl 1990, Benjamin 1995). It was pointed out (Beker et al. 2016; Eberhardt et al. 2020a) that, given the difficulty of species identification within the genus, one could not be certain which toxic compounds referred to which species.

Carrasco-Hernández et al. (2015) described *Hebeloma* spp. obtained from the Ozumba market, thus presumably intended for human consumption. They recognize three different species, identified as *H.alpinum*, *H.leucosarx* and *H.mesophaeum*. These identifications have to be treated with caution. Certainly, the basidiospore measures they give for *H.alpinum* and *H.leucosarx* would appear too small for those species as we interpret them today. The fact that the spore sizes they give for all three species differ considerably from the spore size of *H.ambustiterranum* would suggest that more than one species of *Hebeloma* is consumed in Mexico.

*Hebelomaambustiterranum* is a species of great cultural significance in central Mexico, since it is used as food for the preparation of several local recipes. It is commonly and widely sold in local food markets. Traditional management practices are carried out to encourage the production of basidiomes, such as the use of fire. Traditional names have been assigned to the edible taxa of the genus, and it appears that their distribution is wide. However, the analysis of a far greater number of samples is required before the real diversity of this group of species may be known and the knowledge of the edible mushrooms of Mexico expanded.

*Hebeloma* species have been considered as “early-stage [ectomycorrhizal] fungi” (Deacon et al. 1983; Mason et al. 1983; Gryta et al. 1997) and gained a reputation as nursery fungi (e.g., Castellano and Molina 1989; Menkis and Vasaitis 2011). There are other species in the genus, further to *H.ambustiterranum*, known to associate with burnt ground (Beker et al. 2016). High pH and nutrient levels are associated both with nurseries and burnt ground. It is not clear whether *H.ambustiterranum* occurs in nurseries. However, should *H.ambustiterranum* be considered for nursery typo utilizing edibles, knowing about the fire ecology should be helpful in establishing inoculum production and stabilizing *H.ambustiterranum* populations in the long-term.

While this study was limited with regard to collecting sites and the number of collections studied, nevertheless, with eleven species new to Mexico, it provides an important step in the understanding of the *Hebeloma* of Mexico and a basis for further development. Given how little we know about *Hebeloma* of Mexico, it appears premature to attempt a key. In lieu of a key for *Hebeloma* in Mexico (which would be deficient, based on too few collections), we refer to an interactive identification tool for *Hebeloma* that is currently under development (Bartlett et al. 2021, accepted).

## Supplementary Material

XML Treatment for Hebeloma ambustiterranum

XML Treatment for Hebeloma cohaerens

XML Treatment for Hebeloma magnicystidiatum

XML Treatment for Hebeloma neurophyllum

XML Treatment for Hebeloma subaustrale

## Supplementary materials

Supplementary material 1 Sequences used in the analyse Data type Docx file. Explanation note Sequences used in the analyses. Herbarium abbreviations follow Index Herbariorum (http://sweetgum.nybg.org/science/ih/) and are separated from the specimen numbers by a space or by a hyphen. MuOb, Mushroom Observer https://mushroomobserver.org/. HJB, personal collection of H.J. Beker unless preceded by an herbarium abbreviation. https://binary.pensoft.net/file/708815This dataset is made available under the Open Database License (http://opendatacommons.org/licenses/odbl/1.0/). The Open Database License (ODbL) is a license agreement intended to allow users to freely share, modify, and use this Dataset while maintaining this same freedom for others, provided that the original source and author(s) are credited.Ursula Eberhardt, Alejandro Kong, Adriana Montoya, Nicole Schütz, Peter Bartlett, Henry J. Beker

Supplementary material 2 Alignments and trees Data type Txt file. Explanation note This file includes all alignments and trees, including single locus trees associated with Eberhardt et al. (2022) Not (only) poison pies – *Hebeloma* (Hymenogastraceae, Agaricales) in Mexico. https://binary.pensoft.net/file/708816This dataset is made available under the Open Database License (http://opendatacommons.org/licenses/odbl/1.0/). The Open Database License (ODbL) is a license agreement intended to allow users to freely share, modify, and use this Dataset while maintaining this same freedom for others, provided that the original source and author(s) are credited.Ursula Eberhardt, Alejandro Kong, Adriana Montoya, Nicole Schütz, Peter Bartlett, Henry J. Beker

## References

[B1] Aanen DK Kuyper TW (1999) Intercompatibility tests in the *Hebelomacrustuliniforme* complex in northwestern Europe. Mycologia 91(5): 783–795. 10.1080/00275514.1999.12061084

[B2] Aanen DK Kuyper TW Hoekstra RF (2001) A widely distributed ITS polymorphism within a biological species of the ectomycorrhizal fungus *Hebelomavelutipes*. Mycological Research 105(3): 284–290. 10.1017/S0953756201003628

[B3] Abarenkov K Tedersoo L Nilsson RH Vellak K Saar I Veldre V Parmasto E Prous M Aan A Ots M Kurina O Ostonen I Jõgeva J Halapuu S Põldmaa K Toots M Truu J Larsson K-H Kõljalg U (2010) PlutoF – a Web based workbench for ecological and taxonomic research, with an online implementation for fungal ITS sequences. Evolutionary Bioinformatics Online 6: 189–196. 10.4137/EBO.S6271

[B4] Aremu MO Basu SK Gyar SD Goyal A Bhowmik PK Datta Banik S (2009) Proximate composition and functional properties of mushroom flours from *Ganoderma* spp., *Omphalotusolearius* (DC.) Sing. and *Hebelomamesophaeum* (Pers.) Quél. used in Nasarawa State, Nigeria. Malaysian Journal of Nutrition 15: 233–241. 22691821

[B5] Atkinson GF (1909) Preliminary notes on some new species of Agaricaceae and *Clavaria*. Annales Mycologici 7: 365–376.

[B6] Barroetaveña C Rajchenberg MC (2005) Mycorrhizal fungi in *Pinusponderosa* introduced in Central Patagonia (Argentina). Nova Hedwigia 80(3–4): 453–464. 10.1127/0029-5035/2005/0080-0453

[B7] Bartlett P Eberhardt U Schütz N Beker HJ (2021) Machine learning for species identification: The *Hebeloma* project from database to website. Biodiversity Information Science and Standards 5: e73972. 10.3897/biss.5.73972

[B8] Beker HJ Eberhardt U Vesterholt J (2010) *Hebelomahiemale* Bres. in arctic/alpine habitats. North American Fungi 5: 51–65.

[B9] Beker HJ Eberhardt U Vesterholt J Hawksworth DL (2013) Proposal to conserve the name *Agaricuslaterinus* (*Hebelomalaterinum*) against the sanctioned *Agaricusfastibilis* (*Hebelomafastibile*) (Basidiomycota: Agaricales: Strophariaceae). Taxon 62: 1059–1060. 10.12705/625.27

[B10] Beker HJ Eberhardt U Vesterholt J (2016) *Hebeloma* (Fr.) P. Kumm. Fungi Europaei 13. Edizioni Tecnografica, Lomazzo, Italy, 1232 pp.

[B11] Benjamin DR (1995) Mushrooms, Poisons and Panaceas. W.H. Freeman and Company, New York.

[B12] Bresinsky A Besl H (1990) A Colour Atlas of Poisonous Fungi. Wolfe, London, 295 pp.

[B13] Carrasco-Hernández V Pérez-Moreno J Quintero-Lizaola R Espinosa-Solares T Lorenzana-Fernández A Espinosa-Hernández V (2015) Edible species of the fungal genus *Hebeloma* and two neotropical pines. Pakistan Journal of Botany 47: 319–326.

[B14] Castellano MA Molina R (1989) Mycorrhizae. In: McDonald SE Barnett JP (Eds) The Container Tree Nursery Manual, vol 5 Agricultural Handbook 674.U.S. Department of Agriculture, Forest Service, Washington D.C., 101–167.

[B15] Cripps C Eberhardt U Schütz N Beker HJ Evenson VS Horak E (2019) The genus *Hebeloma* in the Rocky Mountain alpine zone. MycoKeys 46: 1–54. 10.3897/mycokeys.46.32823 PMC6379322 30787668

[B16] Deacon JW Donaldson SJ Last FT (1983) Sequences and interactions of mycorrhizal fungi on birch. Plant and Soil 71(1–3): 257–262. 10.1007/BF02182660

[B17] Eberhardt U (2012) Methods for DNA barcoding fungi. In: Kress JW Erickson DL (Eds) DNA Barcodes: Methods and Protocols.Humana Press Imprint (Springer), New York, 183–205. 10.1007/978-1-61779-591-6_9

[B18] Eberhardt U Beker HJ (2010) *Hebelomavesterholtii*, a new species in section Theobromina. Mycological Progress 9(2): 215–223. 10.1007/s11557-009-0627-z

[B19] Eberhardt U Beker HJ Vila J Vesterholt J Llimona X Gadjieva R (2009) *Hebeloma* species associated with *Cistus*. Mycological Research 113(1): 153–162. 10.1016/j.mycres.2008.09.007 18940258

[B20] Eberhardt U Beker HJ Vesterholt J Dukik K Walther G Vila J Fernández Brime S (2013) European species of HebelomasectionTheobromina. Fungal Diversity 58(1): 103–126. 10.1007/s13225-012-0188-3

[B21] Eberhardt U Beker HJ Vesterholt J (2015) Decrypting the *Hebelomacrustuliniforme* complex: European species of HebelomasectionDenudatasubsectionDenudata. Persoonia 35: 101–147. 10.3767/003158515X687704 26823631 PMC4713102

[B22] Eberhardt U Beker HJ Vesterholt J Schütz N (2016a) The taxonomy of the European species of HebelomasectionDenudata subsections *Hiemalia*, Echinospora subsect. nov. and Clepsydroida subsect. nov. and five new species. Fungal Biology 120(1): 72–103. 10.1016/j.funbio.2015.09.014 26693686

[B23] Eberhardt U Ronikier A Schütz N Beker HJ (2016b) The genus *Hebeloma* in the alpine belt of the Carpathians including two new species. Mycologia 107(6): 1285–1303. 10.3852/15-097 26354807

[B24] Eberhardt U Beker HJ Schütz N Pedersen OS Sysouphanthong P Læssøe T (2020a) Adventurous cuisine in Laos: *Hebelomaparvisporum*, a new species in HebelomasectionPorphyrospora. Mycologia 112(1): 172–184. 10.1080/00275514.2019.1680220 31900082

[B25] Eberhardt U Beker HJ Schütz N Mikami M Kasuya T (2020b) Rooting Hebelomas: The Japanese ‘*Hebelomaradicosum*’ is a distinct species, *Hebelomasagarae* sp. nov. (Hymenogastraceae, *Agaricales*). Phytotaxa 456(2): 125–144. 10.11646/phytotaxa.456.2.1

[B26] Eberhardt U Beker HJ Borgen T Knudsen H Schütz N Elborne SA (2021a) A survey of *Hebeloma* (Hymenogastraceae) in Greenland. MycoKeys 79: 17–118. 10.3897/mycokeys.79.63363 33958950 PMC8076164

[B27] Eberhardt U Schütz N Beker HJ Lee S Horak E (2021b) *Hebeloma* in the Malay Peninsula: Masquerading within *Psathyrella*. MycoKeys 77: 117–141. 10.3897/mycokeys.77.57394 33551660 PMC7862216

[B28] Eberhardt U Schütz N Bartlett P Beker HJ (2022a) 96 North American taxa sorted – Peck’s *Hebeloma* revisited. Mycologia online early. 10.1080/00275514.2021.2012063 35230235

[B29] Eberhardt U Schütz N Bartlett P Hosaka K Kasuya T Beker HJ (2022b) Revisiting *Hebeloma* (Hymenogastraceae, Agaricales) in Japan: Four species recombined into other genera but three new species discovered. Mycological Progress 21(1): 447–472. 10.1007/s11557-021-01757-x

[B30] Estrada-Martínez E Guzmán G Tovar DC Ortega Paczka R (2009) Contribución al conocimiento etnomicológico de los hongos comestibles silvestres de mercados regionales y comunidades de la Sierra Nevada (México). Interciencia 34: 25–33.

[B31] Flores Arzú R (2020) Diversity and importance of edible ectomycorrhizal fungi in Guatemala. In: Pérez-Moreno J Guerin-Laguette A Flores Arzú R Yu F-Q (Eds) Mushrooms, Humans and Nature in a Changing World.Springer, Cham, 101–140. 10.1007/978-3-030-37378-8_4

[B32] Gagné A Jean-Luc Jany J-L Bousquet J Khasa DP (2006) Ectomycorrhizal fungal communities of nursery-inoculated seedlings outplanted on clear-cut sites in northern Alberta. Canadian Journal of Forest Research 36(7): 1684–1694. 10.1139/x06-063

[B33] Garibay-Orijel R Morales-Marañon E Domínguez-Gutiérrey M Flores-García A (2013) Caracterización morfológica y genética de las ectomicorrizas formadas entre *Pinusmontezumae* y los hongos presentes en los bancos de esporas en la Faja Volcánica Transmexicana. Revista Mexicana de Biodiversidad 84(1): 153–169. 10.7550/rmb.29839

[B34] Gonzalez P Labarère J (1998) Sequence and secondary structure of the mitochondrial small-subunit rRNA V4, V6, and V9 domains reveal highly species-specific variations within the genus *Agrocybe*. Applied and Environmental Microbiology 64(11): 4149–4160. 10.1128/AEM.64.11.4149-4160.1998 9797259 PMC106621

[B35] Grilli E Beker HJ Eberhardt U Schütz N Leonardi M Vizzini A (2016) Unexpected species diversity and contrasting evolutionary hypotheses in *Hebeloma* sections *Sinapizantia* and *Velutipes* in Europe. Mycological Progress 15(1): 1–46. 10.1007/s11557-015-1148-6

[B36] Gryta H Debaud J-C Effosse G Marmeisse R (1997) Fine scale structure of populations of the ectomycorrhizal fungus *Hebelomacylindrosporum* in coastal sand dune forest ecosystems. Molecular Ecology 6(4): 353–364. 10.1046/j.1365-294X.1997.00200.x

[B37] Guindon S Dufayard J-F Lefort V Anisimova M Hordijk W Gascuel O (2010) New algorithms and methods to estimate Maximum-Likelihood phylogenies: Assessing the performance of PhyML 3.0. Systematic Biology 59(3): 307–321. 10.1093/sysbio/syq010 20525638

[B38] Guzmán G (1977) Identificación de Los Hongos: Comestibles, Venenosos, Alucinantes y Destructores de La Madera. Editorial Limusa, Mexico City, 236 pp.

[B39] Hesler LR (1977) New species of Hebeloma. Kew Bulletin 31(3): 471–480. 10.2307/4119390

[B40] Hoang DT Chernomor O von Haeseler A Minh BQ Vinh LS (2018) UFBoot2: Improving the ultrafast bootstrap approximation. Molecular Biology and Evolution 35(2): 518–522. 10.1093/molbev/msx281 29077904 PMC5850222

[B41] Kalyaanamoorthy S Minh BQ Wong TKF von Haeseler A Jermiin LS (2017) Fast model selection for accurate phylogenetic estimates. Nature Methods 14(6): 587–589. 10.1038/nmeth.4285 28481363 PMC5453245

[B42] Katoh K Standley DM (2013) MAFFT multiple sequence alignment software version 7: Improvements in performance and usability. Molecular Biology and Evolution 30(4): 772–780. 10.1093/molbev/mst010 23329690 PMC3603318

[B43] Katoh K Kuma K Toh H Miyata T (2005) MAFFT version 5: Improvement in accuracy of multiple sequence alignment. Nucleic Acids Research 33(2): 511–518. 10.1093/nar/gki198 15661851 PMC548345

[B44] Katoh K Rozewicki J Yamada KD (2019) MAFFT online service: Multiple sequence alignment, interactive sequence choice and visualization. Briefings in Bioinformatics 20(4): 1160–1166. 10.1093/bib/bbx108 28968734 PMC6781576

[B45] Kauff F Lutzoni F (2002) Phylogeny of the Gyalectales and Ostropales (Ascomycota, Fungi): Among and within order relationships based on nuclear ribosomal RNA small and large subunits. Molecular Phylogenetics and Evolution 25(1): 138–156. 10.1016/S1055-7903(02)00214-2 12383757

[B46] Kõljalg U Nilsson RH Abarenkov K Tedersoo L Taylor AFS Bahram M Bates ST Bruns TD Bengtsson-Palme J Callaghan TM Douglas B Drenkhan T Eberhardt U Dueñas M Grebenc T Griffith GW Hartmann M Kirk PM Kohout P Larsson E Lindahl BD Lücking R Martín MP Matheny PB Nguyen NH Niskanen T Oja J Peay KG Peintner U Peterson M Põldmaa K Saag L Saar I Schüßler A Scott JA Senés C Smith ME Suija A Taylor DL Telleria MT Weiß M Larsson K-H (2013) Towards a unified paradigm for sequence-based identification of Fungi. Molecular Ecology 22(21): 5271–5277. 10.1111/mec.12481 24112409

[B47] Kõljalg U Nilsson HR Schigel D Tedersoo L Larsson K-H May TW Taylor AFS Jeppesen TS Frøslev TG Lindahl BD Põldmaa K Saar I Suija A Savchenko A Yatsiuk I Adojaan K Ivanov F Piirmann T Pöhönen R Zirk A Abarenkov K (2020) The taxon hypothesis paradigm—On the unambiguous detection and communication of taxa. Microorganisms 8(12): 1910. 10.3390/microorganisms8121910 33266327 PMC7760934

[B48] Kumar S Stecher G Li M Knyaz C Tamura K (2018) MEGA X: Molecular Evolutionary Genetics Analysis across computing platforms. Molecular Ecology and Evolution 35(6): 1547–1549. 10.1093/molbev/msy096 PMC5967553 29722887

[B49] Larsson A (2014) AliView: A fast and lightweight alignment viewer and editor for large data sets. Bioinformatics (Oxford, England) 30(22): 3276–3278. 10.1093/bioinformatics/btu531 25095880 PMC4221126

[B50] Mason PA Wilson J Last FT Walker C (1983) The concept of succession in relation to the spread of sheathing mycorrhizal fungi on inocculated tree seedlings growing in unsterile soils. Plant and Soil 71(1–3): 247–256. 10.1007/BF02182659

[B51] Menkis A Vasaitis R (2011) Fungi in roots of nursery grown *Pinussylvestris*: Ectomycorrhizal colonialization, genetic diversity and spatial distribution. Microbial Ecology 61(1): 52–63. 10.1007/s00248-010-9676-8 20437259

[B52] Minh BQ Nguyen MAT von Haeseler A (2013) Ultrafast approximation for phylogenetic bootstrap. Molecular Biology and Evolution 30(5): 1188–1195. 10.1093/molbev/mst024 23418397 PMC3670741

[B53] Monedero LC Alvarado P (2020) *Hebelomaadherens*: Una nueva especie de la sección Adherentia sect. nov. Yesca 32: 56–67.

[B54] Montoya A Estrada-Torres A Caballero J (2002) Comparative ethonomycological survey of three localities from La Malinche volcano, Mexico. Journal of Ethnobiology 22: 103–133.

[B55] Montoya A Hernández N Mapes C Kong A Estrada-Torres A (2008) The collection and sale of wild mushrooms in a community of Tlaxcala, Mexico. Economic Botany 62(3): 413–424. 10.1007/s12231-008-9021-z

[B56] Murrill WA (1945) More Florida fungi. Lloydia 8: 263–290.

[B57] Nguyen L-T Schmidt HA von Haeseler A Minh BQ (2015) IQ-TREE: A fast and effective stochastic algorithm for estimating maximum likelihood phylogenies. Molecular Biology and Evolution 32(1): 268–274. 10.1093/molbev/msu300 25371430 PMC4271533

[B58] Oliveira RS Franco AR Vosátka M Castro PML (2010) Management of nursery practices for efficient ectomycorrhizal fungi application in the production of *Quercusilex*. Symbiosis 52(2–3): 125–131. 10.1007/s13199-010-0092-0

[B59] Peay KG Bruns TD Kennedy PG Bergemann SE Garbelotto M (2007) A strong species-area relationship for eukaryotic soil microbes: Island size matters for ectomycorrhizal fungi. Ecology Letters 10(6): 470–480. 10.1111/j.1461-0248.2007.01035.x 17498146

[B60] Pérez-Moreno J Martínez-Reyes M Hernández-Santiago F Ortiz-Lopez I (2020) Climate change, biotechnology, and Mexican neotropical edible ectomycorrhizal mushrooms. In: Pérez-Moreno J Guerin-Laguette A Flores Arzú R Yu F-Q (Eds) Mushrooms, Humans and Nature in a Changing World.Springer, Cham, 61–99. 10.1007/978-3-030-37378-8_3

[B61] Pérez-Moreno J Guerin-Laguette A Rinaldi AC Yu F Verbeken A Hernández-Santiago F Martínez-Reyes M (2021) Edible mycorrhizal fungi of the world: What is their role in forest sustainability, food security, biocultural conservation and climate change? Plants People Planet 3(5): 471–490. 10.1002/ppp3.10199

[B62] Reyes-López RC Montoya A Kong A Cruz-Campuzano EA Caballero-Nieto J (2020) Folk classification of wild mushrooms from San Isidro Buensuceso, Tlaxcala, Central Mexico. Journal of Ethnobiology and Ethnomedicine 16(53): 1–21. 10.1186/s13002-020-00408-x 32928249 PMC7488656

[B63] Schoch CL Seifert KA Huhndorf S Robert V Spouge JL Levesque CA Chen W Bolchacova E Voigt K Crous PW Miller AN Wingfield MJ Aime MC An K-D Bai F-Y Barreto RW Begerow D Bergeron M-J Blackwell M Boekhout T Bogale M Boonyuen N Burgaz AR Buyck B Cai L Cai Q Cardinali G Chaverri P Coppins BJ Crespo A Cubas P Cummings C Damm U de Beer ZW de Hoog GS Del-Prado R Dentinger B Diéguez-Uribeondo J Divakar PK Douglas B Dueñas M Duong TA Eberhardt U Edwards JE Elshahed MS Fliegerova K Furtado M García MA Ge Z-W Griffith GW Griffiths K Groenewald JZ Groenewald M Grube M Gryzenhout M Guo L-D Hagen F Hambleton S Hamelin RC Hansen K Harrold P Heller G Herrera C Hirayama K Hirooka Y Ho H-M Hoffmann K Hofstetter V Högnabba F Hollingsworth PM Hong S-B Hosaka K Houbraken J Hughes K Huhtinen S Hyde KD James T Johnson EM Johnson JE Johnston PR Jones EBG Kelly LJ Kirk PM Knapp DG Kõljalg U Kovács GM Kurtzman CP Landvik S Leavitt SD Liggenstoffer AS Liimatainen K Lombard L Luangsa-ard JJ Lumbsch HT Maganti H Maharachchikumbura SSN Martin MP May TW McTaggart AR Methven AS Meyer W Moncalvo J-M Mongkolsamrit S Nagy LG Nilsson RH Niskanen T Nyilasi I Okada G Okane I Olariaga I Otte J Papp T Park D Petkovits T Pino-Bodas R Quaedvlieg W Raja HA Redecker D Rintoul TL Ruibal C Sarmiento-Ramírez JM Schmitt I Schüßler A Shearer C Sotome K Stefani FOP Stenroos S Stielow B Stockinger H Suetrong S Suh S-O Sung G-H Suzuki M Tanaka K Tedersoo L Telleria MT Tretter E Untereiner WA Urbina H Vágvölgyi C Vialle A Vu TD Walther G Wang Q-M Wang Y Weir BS Weiß M White MM Xu J Yahr R Yang ZL Yurkov A Zamora J-C Zhang N Zhuang W-Y Schindel D (2012) Nuclear ribosomal internal transcribed spacer (ITS) region as a universal DNA barcode marker for Fungi. Proceedings of the National Academy of Sciences of the United States of America 109(16): 6241–6246. 10.1073/pnas.1117018109 22454494 PMC3341068

[B64] Schoch CL Robbertse B Robert V Vu D Cardinali G Irinyi L Meyer W Nilsson RH Hughes KW Miller AN Kirk PM Abarenkov K Aime MC Ariyawansa HA Bidartondo MI Boekhout T Buyck B Cai Q Chen J Crespo A Crous PW Damm U De Beer ZW Dentinger BTM Divakar APK Duen M Feau N Fliegerova K Garcıa MA Ge ZW Griffith GW Groenewald JZ Groenewald M Grube M Gryzenhout M Gueidan C Guo L Hambleton S Hamelin R Hansen K Hofstetter V Seung-Beom Hong S-B Houbraken J Hyde KD Inderbitzin P Johnston PR Karunarathna SC Kõljalg U Kovács GM Kraichak E Krizsan K Kurtzman CP Larsson K-H Leavitt S Letcher PM Liimatainen K Liu J-K Lodge DJ Luangsa-ard JJ Lumbsch HT Maharachchikumbura SSN Manamgoda D Martín MP Minnis AM Moncalvo J-M Mulé G Nakasone KK Niskanen T Olariaga I Papp T Petkovits T Pino-Bodas R Powell MJ Raja HA Redecker D Sarmiento-Ramirez JM Seifert KA Shrestha B Stenroos S Stielow B Suh S-O Tanaka K Tedersoo L Telleria MT Dhanushka Udayanga D Untereiner WA Uribeondo JD Subbarao KV (2014) lgyi CV, Visagie C, Voigt K, Walker DM, Weir BS, Weiß M, Wijayawardene NN, Wingfield MJ, Xu JP, Yang ZL, Zhang N, Zhuang W-Y, Federhen S (2014) Finding needles in haystacks: Linking scientific names, reference specimens and molecular data for Fungi. Database (Oxford): 1–21. 10.1093/database/bau061/2634542 PMC4075928 24980130

[B65] Smith AH Evenson VS Mitchel DH (1983) The Veiled Species of *Hebeloma* in the Western United States. University of Michigan Press, Ann Arbor, Michigan, 219 pp. 10.3998/mpub.12590

[B66] Stecher G Tamura K Kumar S (2020) Molecular Evolutionary Genetics Analysis (MEGA) for macOS. Molecular Biology and Evolution 37(4): 1237–1239. 10.1093/molbev/msz312 31904846 PMC7086165

[B67] Tedersoo L Mikryukov V Anslan S Bahram M Khalid AN Corrales A Agan A Vasco-Palacios A-M Saitta A Antonelli A Rinaldi AC Verbeken A Sulistyo BP Tamgnoue B Furneaux B Ritter CD Nyamukondiwa C Sharp C Marín C Dai DQ Gohar D Sharmah D Biersma EM Cameron EK De Crop E Otsing E Davydov EA Albornoz FE Brearley FQ Buegger F Gates G Zahn G Bonito G Hiiesalu I Hiiesalu I Zettur I Barrio IC Pärn J Heilmann-Clausen J Ankuda J Kupagme JY Sarapuu J Maciá-Vicente JG Fovo JD Geml J Alatalo JM Alvarez-Manjarrez J Monkai J Põldmaa K Runnel K Adamson K Bråthen KA Pritsch K Tchan KI Armolaitis K Hyde KD Newsham KK Panksep K Adebola LA Lamit LJ Saba M da Silva Cáceres ME Tuomi M Gryzenhout M Bauters M Bálint M Wijayawardene N Hagh-Doust N Yorou NS Kurina O Mortimer PE Meidl P Nilsson RH Puusepp R Casique-Valdés R Drenkhan R Garibay-Orijel R Godoy R Alfarraj S Rahimlou S Põlme S Dudov SV Mundra S Ahmed T Netherway T Henkel TW Roslin T Fedosov VE Onipchenko VG Yasanthika WAE Lim YW Piepenbring M Klavina D Kõljalg U Abarenkov K (2021) The Global Soil Mycobiome consortium dataset for boosting fungal diversity research. Fungal Diversity 111(1): 573–588. 10.1007/s13225-021-00493-7

[B68] Trifinopoulos J Nguyen LT von Haeseler A Minh B (2016) W-IQ-TREE: A fast online phylogenetic tool for maximum likelihood analysis. Nucleic Acids Research 44(W1): W232–W235. 10.1093/nar/gkw256 PMC4987875 27084950

[B69] Vesterholt J (2005) The Genus *Hebeloma*. Fungi of Northern Europe 3. Svampetryk, Tilst, Denmark, 146 pp.

[B70] Viveros-Assad LJ Flores-Encarnación M Carreño-López R Munguía-Pérez R Santiesteban NA García-García SMC (2019) Etnomicología de la Sierra Nevada. RD ICUAP 5(15). http://rd.buap.mx/ojs-dm/index.php/rdicuap/article/view/393 [accessed 19 Jan 2021]

