# Supplementary material for: ﻿Not (only) poison pies – Hebeloma (Agaricales, Hymenogastraceae) in Mexico
Source: MycoKeys. 2022 Jun 30;90:163–202. doi: 10.3897/mycokeys.90.85267 (PMC9849069; doi:10.3897/mycokeys.90.85267)
Supplement: Supplementary material 1 — Sequences used in the analyse [file mycokeys-90-163-s001.docx]

**Supplementary Table 1**. Sequences used in the analyses. Herbarium abbreviations follow Index Herbariorum (<http://sweetgum.nybg.org/science/ih/>) and are separated from the specimen numbers by a space or by a hyphen. MuOb, Mushroom Observer <https://mushroomobserver.org/>. HJB, personal collection of H.J. Beker unless preceded by an herbarium abbreviation.

| Species | Collection acc. no. | Database ref. | Country | *Hebeloma* sect. | ITS GenBank acc. no. | *Tef1a* Genbank acc. no. | *RPB2* Genbank acc. no. | *MCM7* GenBank acc. no. | mitSSU V6 Genbank acc. no. | mitSSU V9 Genbank acc. no. |
| --- | --- | --- | --- | --- | --- | --- | --- | --- | --- | --- |
| *Hebeloma aanenii* | HJB10435 | HJB10435 | UK | *Denudata* | KM390531, KM390532 |  |  |  | ON202580 | KM390192 |
| *H. aanenii* | PDD 102994 | HJB10692 | New Zealand | *Denudata* | JN943874 |  |  |  | ON202579 | KM390208 |
| *H. aanenii* | BR-MYCO 173987-66 (holotype) | HJB12630 | Poland | *Denudata* | KM390723 |  |  |  | KM390478 | KM390308 |
| *H. aanenii* | TLXM HJB16740 | HJB16740 | Mexico | *Denudata* | ON202494 |  |  |  |  | will follow |
| *H. aanenii* | TLXM HJB16741 | HJB16741 | Mexico | *Denudata* | ON202495 |  |  |  | ON202587 | will follow |
| *H. aanenii* | TLXM HJB16742 | HJB16742 | Mexico | *Denudata* | ON202496 |  |  |  | ON202588 |  |
| *H. aanenii* | TLXM HJB16745 | HJB16745 | Mexico | *Denudata* | ON202497 |  |  |  | ON202589 |  |
| *H. alpinum* | HJB11132 | HJB11132 | Switzerland | *Denudata* | KM390590 |  |  |  | KM390387 | KM390226 |
| *H. alpinum* | HJB12005 | HJB12005 | Svalbard | *Denudata* | KM390696 |  |  |  | KM390464 | KM390290 |
| *H. alpinum* | MONT CLC2855 | HJB15331 | USA | *Denudata* | MK281073 |  |  |  | ON202581 | will follow |
| *H. ammophilum* | G EL281-08 | HJB12374 | Hungary | *Denudata* | KT217509 |  |  |  | KT217297 | KT217110 |
| *H. ammophilum* | E. Ludwig personal collection J886I | HJB14471 | Hungary | *Denudata* | MZ782104 |  |  |  | MZ782867 | will follow |
| *H. ammophilum* | E. Bizio personal collection s.n. | HJB15417 | Italy | *Denudata* | MZ782106 |  |  |  | MZ782868 | will follow |
| *H. ammophilum* | E. Grilli personal collection 101029.01 | HJB16438 | Italy | *Denudata* | MZ782140 |  |  |  | MZ782869 | will follow |
| *H. ammophilum* | E. Grilli personal collection 121019.01 | HJB16439 | Italy | *Denudata* | MZ782141 |  |  |  | MZ782870 | will follow |
| *H. cavipes* | HJB9433 | HJB9433 | Spain | *Denudata* | KT217362 |  |  |  | KT217158 | KT217017 |
| *H. cavipes* | HJB10537 | HJB10537 | France | *Denudata* | KT217402 |  |  |  | KT217198 | KT217050 |
| *H. cavipes* | HJB13227 | HJB13227 | Poland | *Denudata* | MZ782102 |  |  |  | MZ782873 | will follow |
| *H. cavipes* | HJB14374 | HJB14374 | USA | *Denudata* | MZ782103 |  |  |  | MZ782875 | will follow |
| *H. cavipes* | N. Siegel personal collection 3537 | HJB18872 | USA | *Denudata* | MZ782148 |  |  |  | MZ782874 | will follow |
| *H. cinnamomeum* | TNS-F-82067 (holotype) | HJB15837 | Japan | *Denudata* | MZ782111 |  |  |  | MZ782879 | will follow |
| *H. cinnamomeum* | Henry J. Beker personal collection TK- B3265 | HJB15838 | Japan | *Denudata* | MZ782112 |  |  |  | MZ782880 | will follow |
| *H. cinnamomeum* | TNS-F-59098 | HJB16236 | Japan | *Denudata* | MZ782128 |  |  |  | MZ782876 | will follow |
| *H. cinnamomeum* | TNS-F-73984 | HJB16245 | Japan | *Denudata* | MZ782134 |  |  |  | MZ782877 | will follow |
| *H. cinnamomeum* | TNS-F-74039 | HJB16246 | Japan | *Denudata* | MZ782135 |  |  |  | MZ782878 | will follow |
| *H. crustuliniforme* | HJB9053 | HJB9053 | Channel Islands | *Denudata* | KF309398, KF309399 |  |  |  | KM390339 | KM390190 |
| *H. crustuliniforme* | HJB11237 | HJB11237 | Spain | *Denudata* | JN943870 |  |  |  | KM390380 | KM390219 |
| *H. crustuliniforme* | L WAG-W9691 | HJB12803 | Netherlands | *Denudata* | KF309414 |  |  |  | KM390447 | KM390274 |
| *H. crustuliniforme* | L WAG-W9581 | HJB12807 | Netherlands | *Denudata* | KF309415 |  |  |  | KM390452 | KM390278 |
| *H. crustuliniforme* | BR MYCO 173989-68 (epitype) | HJB13713 | France | *Denudata* | KF309424 |  |  |  | KM390510 | KM390327 |
| *H. eburneum* | HJB10290 | HJB10290 | Belgium | *Denudata* | KM390533, KM390534 |  |  |  | KM390341 | KT217034 |
| *H. eburneum* | HJB12670 | HJB12670 | Poland | *Denudata* | KM390727, KM390728 |  |  |  | KM390482 | KM390313 |
| *H. eburneum* | TLXM AK4010 | HJB16758 | Mexico | *Denudata* | ON202523 |  |  |  | ON202594 | will follow |
| *H. eburneum* | TLXM AK4022 | HJB16760 | Mexico | *Denudata* | ON202524 |  |  |  | ON202595 | will follow |
| *H. eburneum* | TLXM AK4163 | HJB16761 | Mexico | *Denudata* | ON202525 |  |  |  | ON202596 | will follow |
| *H. eburneum* | TLXM AK3234 | HJB16775 | Mexico | *Denudata* | ON202521 |  |  |  | ON202592 | will follow |
| *H. eburneum* | TLXM AET2356 | HJB16786 | Mexico | *Denudata* | ON202519 |  |  |  | ON202590 | will follow |
| *H. eburneum* | TLXM AK1373 | HJB16792 | Mexico | *Denudata* | ON202520 |  |  |  | ON202591 | will follow |
| *H. eburneum* | TLXM AK3551 | HJB16796 | Mexico | *Denudata* | ON202522 |  |  |  | ON202593 | will follow |
| *H. eburneum* | DUKE 0351128 | HJB17317 | USA | *Denudata* | MT157296 |  |  |  | ON202582 | will follow |
| *H. eburneum* | C-F-111117 | HJB18936 | Greenland | *Denudata* | MW445590 |  |  |  | ON202584 | will follow |
| *H. echinosporum* | BR-MYCO 174907-16 (holotype) | HJB13524 | France | *Denudata* | KT217548 |  |  |  | KT217330 | KT217132 |
| *H. geminatum* | C-F-90152 (holotype) | HJB10833 | Denmark | *Denudata* | KF309405 |  |  |  | KM390361 | KM390213 |
| *H. geminatum* | TURA 17928F | HJB10961 | Estonia | *Denudata* | KM390583 |  |  |  | KM390381 | KM390220 |
| *H. geminatum* | HJB11801 | HJB11801 | France | *Denudata* | JN943860 |  |  |  | KM390409 | KM390249 |
| *H. hiemale* | HJB8890 | HJB8890 | Belgium | *Denudata* | GQ869484 |  |  |  | KT217148 | KT217009 |
| *H. hiemale* | HJB9384 | HJB9384 | UK | *Denudata* | GQ869482 |  |  |  | KT217156 | KT217015 |
| *H. hiemale* | BR-MYCO 174905-14 (epitype) | HJB11704 | France | *Denudata* | GQ869505 |  |  |  | KT217279 | KT217101 |
| *H. hiemale* | DUKE 0351151 | HJB17319 | USA | *Denudata* | MT157298 |  |  |  | ON202583 | will follow |
| *H. hiemale* | C-F-106776 | HJB17443 | Greenland | *Denudata* | MW445625 |  |  |  | MZ782881 | will follow |
| *H. hiemale* | C-F-106777 | HJB17446 | Greenland | *Denudata* | MW445626 |  |  |  | ON202585 | will follow |
| *H. hiemale* | HJB17933 | HJB17933 | Canada | *Denudata* | ON202530 |  |  |  | ON202597 | will follow |
| *H. hiemale* | HJB18119 | HJB18119 | Canada | *Denudata* | ON202531 |  |  |  | ON202598 | will follow |
| *H. ingratum* | HJB11311 | HJB11311 | Belgium | *Denudata* | KT217460 |  |  |  | KT217256 | KT217089 |
| *H. ingratum* | L WAG-W9573 | HJB12534 | Netherlands | *Denudata* | KT217517 |  |  |  | KT217307 | KT217117 |
| *H. ingratum* | TLXM AK3373 | HJB16771 | Mexico | *Denudata* | ON202532 |  |  |  | ON202599 | will follow |
| *H. ingratum* | HJB16994 | HJB16994 | USA | *Denudata* | ON202533 |  |  |  | ON202600 | will follow |
| *H. ingratum* | LY BR64-24 (holotype) | HJB1000040 | France | *Denudata* | KT217570 |  |  |  | KT217347 | KT217138 |
| *H. limbatum* | HJB9423 | HJB9423 | Spain | *Denudata* | KT217361 |  |  |  | KT217157 | KT217016 |
| *H. limbatum* | C-F-92311 (holotype) | HJB11858 | Italy | *Denudata* | KT217490 |  |  |  | KT217287 | KT217105 |
| *H. limbatum* | L WAG-W9574 | HJB12535 | Netherlands | *Denudata* | KT217518 |  |  |  | KT217308 | KT217118 |
| *H. limbatum* | G. Corriol personal collection 99 10 18 02 | HJB12942 | France | *Denudata* | KT217530 |  |  |  | KT217318 | KT217123 |
| *H. magnicystidiatum* | TLXM 6157 (holotype) | HJB16795 | Mexico | *Denudata* | ON202534 |  |  |  | ON202601 | will follow |
| *H. matritense* | BR-MYCO 174910-19 (holotype) | HJB9485 | Spain | *Denudata* | KT217364 |  |  |  | KT217160 | KT217018 |
| *H. matritense* | E. Grilli personal collection 091112.05 | HJB15672 | Italy | *Denudata* | ON202535 |  |  |  | ON202602 | will follow |
| *H. populinum* | HJB13758 | HJB13758 | UK | *Denudata* | KT217560 |  |  |  | KT217341 | KT217136 |
| *H. populinum* | G. Konstantinidis personal collection 5803 | HJB14114 | Greece | *Denudata* | KT217563 |  |  |  | KT217342 | KT217137 |
| *H. sordidulum* | HJB12269 | HJB12269 | USA | *Denudata* | MZ782101 |  |  |  | MZ782883 | will follow |
| *H. sordidulum* | HJB12287 | HJB12287 | USA | *Denudata* | MZ782100 |  |  |  | MZ782884 | will follow |
| *H. sordidulum* | D.P. Lewis personal collection 11915 | HJB15700 | USA | *Denudata* | MZ782107 |  |  |  | MZ782885 | will follow |
| *H. sordidulum* | MuOb231249 (UWO) | HJB15800 | Canada | *Denudata* | MZ782108 |  |  |  | MZ782882 | will follow |
| *H. sordidulum* | TLXM AK3773 | HJB16754 | Mexico | *Denudata* | ON202559 |  |  |  | ON202609 | will follow |
| *H. sordidulum* | HJB16978 | HJB16978 | USA | *Denudata* | MZ782143 |  |  |  | MZ782886 | will follow |
| *H. vaccinum* | HJB9965 | HJB9965 | Belgium | *Denudata* | KT217371 |  |  |  | KT217167 | KT217023 |
| *H. vaccinum* | HJB10067 | HJB10067 | Belgium | *Denudata* | KT217373 |  |  |  | KT217169 | KT217025 |
| *H. vaccinum* | MONT CLC1881 | HJB15327 | USA | *Denudata* | MK281113 |  |  |  | MZ782889 | will follow |
| *H. vaccinum* | DUKE 0351073 | HJB17310 | USA | *Denudata* | MT157290 |  |  |  | MZ782888 | will follow |
| *H. vaccinum* | C-F-106767 | HJB17492 | Greenland | *Denudata* | MW445830 |  |  |  | MZ782887 | will follow |
| *H. alpinicola* | HJB11019 | HJB11019 | Iceland | *Hebeloma* | ON202500 | ON167790 | ON167837 |  |  |  |
| *H. alpinicola* | HJB12439 | HJB12439 | USA | *Hebeloma* | MK281065 | ON167777 | ON167848 |  |  |  |
| *H. alpinicola* | HJB15611 | HJB15611 | Georgia | *Hebeloma* | ON202499 | ON167789 | ON167858 |  |  |  |
| *H. alpinicola* | MICH 5549 (holotype) | HJB1000311 | USA | *Hebeloma* | MK280987 |  |  |  |  |  |
| *H. ambustiterranum* | TLXM HJB16748 | HJB16748 | Mexico | *Hebeloma* | ON202502 | ON167792 | ON167869 |  |  |  |
| *H. ambustiterranum* | TLXM HJB16750 | HJB16750 | Mexico | *Hebeloma* | ON202503 | ON167793 | ON167870 |  |  |  |
| *H. ambustiterranum* | TLXM HJB16753 | HJB16753 | Mexico | *Hebeloma* | ON202504 | ON167794 | ON167871 |  |  |  |
| *H. ambustiterranum* | TLXM HJB16799 | HJB16799 | Mexico | *Hebeloma* | ON202505 | ON167795 | ON167874 |  |  |  |
| *H. ambustiterranum* | TLXM 6155 (holotype) | HJB16802 | Mexico | *Hebeloma* | ON202501 | ON167791 | ON167875 |  |  |  |
| *H. ambustiterranum* | TLXM HJB16803 | HJB16803 | Mexico | *Hebeloma* | ON202506 | ON167796 | ON167876 |  |  |  |
| *H. ambustiterranum* | TLXM HJB16805 | HJB16805 | Mexico | *Hebeloma* | ON202507 | ON167797 | ON167877 |  |  |  |
| *H. colvinii* | C-F-104038 | HJB17684 | Greenland | *Hebeloma* | MW445747 | ON167784 | ON167887 |  |  |  |
| *H. colvinii* | C-F-104035 | HJB17685 | Greenland | *Hebeloma* | MW445748 | ON167785 | ON167888 |  |  |  |
| *H. colvinii* | DAOM 231984 | HJB19415 | Canada | *Hebeloma* | ON202513 | ON167798 | ON167896 |  |  |  |
| *H. colvinii* | C-F-107346 | HJB19653 | Greenland | *Hebeloma* | MW445749 | ON167786 | ON167897 |  |  |  |
| *H. colvinii* | NYS-F-000813.1 (lectotype) | HJB1000269 | USA | *Hebeloma* | MN017797 |  |  |  |  |  |
| *H. excedens* | A. Gerenday personal collection 20846 | HJB15702 | USA | *Hebeloma* | ON202528 | ON167806 | ON167860 |  |  |  |
| *H. excedens* | TLXM HJB16734 | HJB16734 | Mexico | *Hebeloma* | ON202526 | ON167804 | ON167866 |  |  |  |
| *H. excedens* | TLXM HJB16817 | HJB16817 | Mexico | *Hebeloma* | ON202527 | ON167805 | ON167880 |  |  |  |
| *H. excedens* | HJB16980 | HJB16980 | USA | *Hebeloma* | ON202529 | ON167807 | ON167882 |  |  |  |
| *H. excedens* | NYS-F-001123 (holotype) | HJB1000268 | USA | *Hebeloma* | MK280986 |  |  |  |  |  |
| *H. marginatulum* | C KK-140202 | HJB10937 | Estonia | *Hebeloma* | MK305912 | ON167780 | ON167836 |  |  |  |
| *H. marginatulum* | HJB11151 | HJB11151 | Switzerland | *Hebeloma* | KT071029 | KT071091 | KT071069 |  |  |  |
| *H. marginatulum* | HJB12009 | HJB12009 | Svalbard | *Hebeloma* | KT071030 | KT071092 | KT071070 |  |  |  |
| *H. marginatulum* | HJB12458 | HJB12458 | USA | *Hebeloma* | MK281064 | ON167776 | ON167849 |  |  |  |
| *H. marginatulum* | KRAM F-46901 | HJB13119 | Poland | *Hebeloma* | KT071028 | KT071090 | KT071068 |  |  |  |
| *H. marginatulum* | C-F-104111 | HJB17683 | Greenland | *Hebeloma* | MW445728 | ON167782 | ON167886 |  |  |  |
| *H. mesophaeum* | HJB10166 | HJB10166 | Belgium | *Hebeloma* | KT218250 | KT217611 | KT217700 |  |  |  |
| *H. mesophaeum* | HJB10683 | HJB10683 | UK | *Hebeloma* | KT218307 | KT217641 | KT217726 |  |  |  |
| *H. mesophaeum* | C JV-03-547 (epitype) | HJB10854 | Denmark | *Hebeloma* | EF451057 |  |  |  |  |  |
| *H. mesophaeum* | HJB11050 | HJB11050 | Iceland | *Hebeloma* | MK961995 | ON167781 | ON167838 |  |  |  |
| *H. mesophaeum* | HJB11944 | HJB11944 | Svalbard | *Hebeloma* | ON202539 | ON167811 | ON167842 |  |  |  |
| *H. mesophaeum* | HJB12114 | HJB12114 | France | *Hebeloma* | ON202537 | ON167809 | ON167847 |  |  |  |
| *H. mesophaeum* | SWGC GM14B-025 | HJB14858 | Canada | *Hebeloma* | ON202536 | ON167808 | ON167857 |  |  |  |
| *H. mesophaeum* | TLXM HJB16737 | HJB16737 | Mexico | *Hebeloma* | ON202538 | ON167810 | ON167867 |  |  |  |
| *H. mesophaeum* | C-F-104301 | HJB17068 | Greenland | *Hebeloma* | MW445741 | ON167783 | ON167884 |  |  |  |
| *H. nigellum* | HJB9516 | HJB9516 | UK | *Hebeloma* | MK281024 | ON167775 | ON167898 |  |  |  |
| *H. nigellum* | C KK-010202 | HJB10936 | Estonia | *Hebeloma* | MK280994 | ON167774 | ON167835 |  |  |  |
| *H. nigellum* | MONT CLC3614b | HJB17305 | USA | *Hebeloma* | MK281071 | ON167778 | ON167885 |  |  |  |
| *H. nigellum* | LY BR66-71 (holotype) | HJB1000042 | France | *Hebeloma* | KX765786 |  |  |  |  |  |
| *H. pascuense* | WTU-F-043852 (holotype) | HJB1000556 | USA | *Hebeloma* | MZ019466 |  |  |  |  |  |
| *H. psammophilum* | S.A. Elborne personal collection 1488 | HJB10653 | Denmark | *Hebeloma* | MZ019467 |  |  |  |  |  |
| *H. psammophilum* | C JV-91-873 | HJB10943 | Denmark | *Hebeloma* | ON202551 | ON167817 |  |  |  |  |
| *H. psammophilum* | LIP PAM08103101 | HJB12970 | France | *Hebeloma* | ON202553 | ON167819 | ON167852 |  |  |  |
| *H. psammophilum* | HJB13337 | HJB13337 | France | *Hebeloma* | ON202552 | ON167818 | ON167853 |  |  |  |
| *H. pubescens* | HJB11932 | HJB11932 | Svalbard | *Hebeloma* | KY271841 | ON167773 | ON167840 |  |  |  |
| *H. pubescens* | BR 5020184127626 (holotype) | HJB12008 | Svalbard | *Hebeloma* | KX765792 | ON167769 | ON167843 |  |  |  |
| *H. pubescens* | HJB12055 | HJB12055 | Svalbard | *Hebeloma* | KY271838 | ON167772 | ON167845 |  |  |  |
| *H. pubescens* | HJB12057 | HJB12057 | Svalbard | *Hebeloma* | KY271837 | ON167771 | ON167846 |  |  |  |
| *H. pubescens* | HJB16886 | HJB16886 | Canada | *Hebeloma* | ON202554 | ON167820 | ON167881 |  |  |  |
| *H. sordescens* | HJB10110 | HJB10110 | UK | *Hebeloma* | ON202558 | ON167822 | ON167830 |  |  |  |
| *H. sordescens* | HJB12627 | HJB12627 | Poland | *Hebeloma* | ON202557 | ON167821 | ON167850 |  |  |  |
| *H. sordescens* | HJB13595 | HJB13595 | Germany | *Hebeloma* | ON202556 |  | ON167854 |  |  |  |
| *H. sordescens* | C JV-84-1371 (holotype) | HJB1000129 | Denmark | *Hebeloma* | KX765787 |  |  |  |  |  |
| *H. subtortum* | HJB10698 | HJB10698 | UK | *Hebeloma* | ON202565 | ON167825 | ON167831 |  |  |  |
| *H. subtortum* | C JV-03-848 | HJB12925 | Italy | *Hebeloma* | ON202563 | ON167823 | ON167851 |  |  |  |
| *H. subtortum* | HJB13784 | HJB13784 | Spain | *Hebeloma* | ON202564 | ON167824 | ON167855 |  |  |  |
| *H. subtortum* | TNS-F-61666 | HJB16241 | Japan | *Hebeloma* | MZ782131 | ON167788 | ON167862 |  |  |  |
| *H. subtortum* | H 4006 (lectotype) | HJB1000157 | Estonia | *Hebeloma* | KX765791 |  |  |  |  |  |
| *H. velatum* | HJB10744 | HJB10744 | UK | *Hebeloma* | ON202518 | ON167803 | ON167832 |  |  |  |
| *H. velatum* | UPS AT2004221 | HJB10779 | Sweden | *Hebeloma* | ON202516 | ON167801 | ON167833 |  |  |  |
| *H. velatum* | HJB10922 | HJB10922 | Belgium | *Hebeloma* | MK305910 | ON167779 | ON167834 |  |  |  |
| *H. velatum* | HJB11093 | HJB11093 | Switzerland | *Hebeloma* | ON202517 | ON167802 | ON167839 |  |  |  |
| *H. velatum* | HJB11936 | HJB11936 | Svalbard | *Hebeloma* | ON202514 | ON167799 | ON167841 |  |  |  |
| *H. velatum* | HJB12017 | HJB12017 | Svalbard | *Hebeloma* | ON202515 | ON167800 | ON167844 |  |  |  |
| *H. velatum* | A. de Haan personal collection 11031 | HJB14141 | Belgium | *Hebeloma* | KY271835 | ON167770 | ON167856 |  |  |  |
| *H. velatum* | NYS-F-003339 (holotype) | HJB1000092 | USA | *Hebeloma* | MN017802 |  |  |  |  |  |
| *H. angustisporium* | TENN-F-023364 (holotype) | HJB1000314 | USA | *Naviculispora* | ON202508 |  |  |  |  |  |
| *H. avellaneum* | SWGC S. May s.n. | HJB14320 | Canada | *Naviculispora* | MK281019 |  |  |  |  |  |
| *H. avellaneum* | DBG-F-020434 | HJB15496 | USA | *Naviculispora* | MK281025 |  |  |  |  |  |
| *H. avellaneum* | DBG-F-019533 | HJB15525 | USA | *Naviculispora* | MK281026 |  |  |  |  |  |
| *H. avellaneum* | MICH 10722 (holotype) | HJB1000322 | USA | *Naviculispora* | MK280988 |  |  |  |  |  |
| *H. catalaunicum* | BR 5020184132484 (holotype) | HJB11519 | Spain | *Naviculispora* | KX765794 |  |  |  |  |  |
| *H. catalaunicum* | MCVE 13641 | HJB14345 | Spain | *Naviculispora* | MK281020 |  |  |  |  |  |
| *H. catalaunicum* | E. Grilli personal collection 051127.01 | HJB14626 | Italy | *Naviculispora* | MK281021 |  |  |  |  |  |
| *H. islandicum* | BR 5020184116583 (holotype) | HJB11034 | Iceland | *Naviculispora* | KX765799 |  |  |  |  |  |
| *H. islandicum* | C-F-103573 | HJB16632 | Greenland | *Naviculispora* | MW445901 |  |  |  |  |  |
| *H. nanum* | HJB11153 | HJB11153 | UK | *Naviculispora* | MK280996 |  |  |  |  |  |
| *H. nanum* | HJB13671 | HJB13671 | Italy | *Naviculispora* | MK281018 |  |  |  |  |  |
| *H. nanum* | TNS-F- 55100 | HJB16232 | Japan | *Naviculispora* | MZ782125 |  |  |  |  |  |
| *H. nanum* | HJB18171 | HJB18171 | Canada | *Naviculispora* | ON202540 |  |  |  |  |  |
| *H. nanum* | PRM153761 (holotype) | HJB1000155 | Czechia | *Naviculispora* | KX765798 |  |  |  |  |  |
| *H. naviculosporum* | HJB13807 | HJB13807 | Germany | *Naviculispora* | KT071039 |  |  |  |  |  |
| *H. naviculosporum* | L. Ballester 11081701 | HJB14211 | Spain | *Naviculispora* | KT071040 |  |  |  |  |  |
| *H. naviculosporum* | KRAM F-57436 | HJB14568 | Slovacia | *Naviculispora* | KT071041 |  |  |  |  |  |
| *H. naviculosporum* | AH 14256 (holotype) | HJB1000023 | Spain | *Naviculispora* | KX765797 |  |  |  |  |  |
| *H. perangustisporium* | TENN-F-013890 | HJB1000450 | USA | *Naviculispora* | ON202548 |  |  |  |  |  |
| *H. pungens* | MICH 5560 | HJB1000474 | USA | *Naviculispora* | ON202555 |  |  |  |  |  |
| *H. subaustrale* | TLXM HC1155 | HJB16793 | Mexico | *Naviculispora* | ON202560 |  |  |  |  |  |
| *H. subaustrale* | D. Bartholow personal collection SPFS-2011-63 | HJB17796 | USA | *Naviculispora* | ON202561 |  |  |  |  |  |
| *H. subaustrale* | HJB18418 | HJB18418 | USA | *Naviculispora* | ON202562 |  |  |  |  |  |
| *H. bulbiferum* | HJB10300 | HJB10300 | Italy | *Sinapizantia* | KT218261 | KT217615 | KT217704 |  | KT218027 |  |
| *H. bulbiferum* | C JV-03-587 | HJB10812 | Italy | *Sinapizantia* | KT218318 | KT217645 | KT217730 |  | KT218074 |  |
| *H. bulbiferum* | E. Campo personal collection 177060 | HJB13083 | Croatia | *Sinapizantia* | KT218422 | KT217667 | KT217754 |  | KT218151 |  |
| *H. sinapizans* | HJB10309 | HJB10309 | italy | *Sinapizantia* | JQ751187 |  | JQ751078 | JQ751118 | KT218029 |  |
| *H. sinapizans* | HJB10360 | HJB10360 | Belgium | *Sinapizantia* | JQ768359 | KT217627 | JQ751077 | JQ751116 | KT218042 |  |
| *H. sinapizans* | HJB10628 | HJB10628 | UK | *Sinapizantia* | JQ751191 | KT217637 | KT217721 | JQ751119 | KT218057 |  |
| *H. sinapizans* | BR 5020184118648 (epitype) | HJB13530 | France | *Sinapizantia* | KT218440 | KT217671 | KT217763 |  | KT218167 |  |
| *H. alboerumpens* | J. Vila personal collection 1090114-15 | HJB13021 | Spain | *Theobromina* | JQ751220 |  | JQ751062 | JQ751104 |  |  |
| *H. alboerumpens* | AH 46601 | HJB15863 | Spain | *Theobromina* | ON202498 |  | ON167861 | ON168960 |  |  |
| *H. alboerumpens* | LIP JVG1080108-19 (holotype) | HJB1000145 | Spain | *Theobromina* | JQ751217 |  | JQ751061 | JQ751103 |  |  |
| *H. cohaerens* | TLXM GF1866 | HJB16779 | Mexico | *Theobromina* | ON202512 |  | ON167872 | ON168964 |  |  |
| *H. cohaerens* | TLXM AME3101 | HJB17732 | Mexico | *Theobromina* | ON202510 |  | ON167889 | ON168962 |  |  |
| *H. cohaerens* | TLXM 6156 (holotype) | HJB17733 | Mexico | *Theobromina* | ON202511 |  | ON167890 | ON168963 |  |  |
| *H. cohaerens* | TLXM AK17-08 | HJB17737 | Mexico | *Theobromina* | ON202509 |  | ON167891 | ON168961 |  |  |
| *H. erumpens* | C JV-05-740 | HJB11407 | Spain | *Theobromina* | EU570185 |  | JQ751066 | JQ751107 |  |  |
| *H. erumpens* | C JV-05-746 | HJB11413 | Spain | *Theobromina* | EU570186 |  | JQ751067 | JQ751108 |  |  |
| *H. erumpens* | J. Vila personal collection 1071029-8 | HJB12250 | Spain | *Theobromina* | JQ751207 |  | JQ751068 | JQ751109 |  |  |
| *H. griseopruinatum* | HJB10897 | HJB10897 | Germany | *Theobromina* | EU570183 |  | JQ751069 | JQ751110 |  |  |
| *H. griseopruinatum* | C-F-89926 (holotype) | HJB13422 | Denmark | *Theobromina* | JQ751183 |  | JQ751071 | JQ751111 |  |  |
| *H. parvicystidiatum* | HJB9405 | HJB9405 | Spain | *Theobromina* | JQ751184 |  | JQ751072 | JQ751112 |  |  |
| *H. parvicystidiatum* | HJB10311 | HJB10311 | Italy | *Theobromina* | JQ751189 |  | JQ751073 | JQ751113 |  |  |
| *H. parvicystidiatum* | C-F-89925 (holotype) | HJB11859 | Italy | *Theobromina* | JQ751205 |  | JQ751074 | JQ751114 |  |  |
| *H. plesiocistum* | LIP JVG1021214-5 | HJB11514 | Spain | *Theobromina* | EU570170 |  | JQ751075 | JQ751115 |  |  |
| *H. plesiocistum* | HJB11868 | HJB11868 | Italy | *Theobromina* | EU570172 |  | JQ751076 | ON168958 |  |  |
| *H. plesiocistum* | E. Grilli personal collection 111127.01 | HJB16472 | Italy | *Theobromina* | ON202549 |  | ON167863 | ON168965 |  |  |
| *H. plesiocistum* | E. Grilli personal collection 151114.08 | HJB16474 | Italy | *Theobromina* | ON202550 |  | ON167864 | ON168966 |  |  |
| *H. theobrominum* | HJB9293 | HJB9293 | UK | *Theobromina* | EU570180 |  | JQ751086 | ON168959 |  |  |
| *H. theobrominum* | HJB10009 | HJB10009 | Estonia | *Theobromina* | EU570181 |  | JQ751087 | JQ751124 |  |  |
| *H. theobrominum* | HJB10063 | HJB10063 | Belgium | *Theobromina* | FJ816623 |  | JQ751089 | JQ751125 |  |  |
| *H. theobrominum* | C HJB11377 | HJB11382 | Spain | *Theobromina* | FJ816624 |  | JQ751090 | JQ751126 |  |  |
| *H. vesterholtii* | HJB10339 | HJB10339 | Italy | *Theobromina* | FJ816629, FJ816630 |  | JQ751099 | JQ751132 |  |  |
| *H. vesterholtii* | HJB10674 | HJB10674 | UK | *Theobromina* | FJ943237, FJ943238 |  | JQ751100 | JQ751133 |  |  |
| *H. vesterholtii* | HJB11377 | HJB11377 | Spain | *Theobromina* | FJ816635 |  | JQ751101 | JQ751134 |  |  |
| *H. vesterholtii* | HJB11869 | HJB11869 | Italy | *Theobromina* | FJ943239, FJ943240 |  | JQ751102 | JQ751135 |  |  |
| *H. vesterholtii* | BR-MYCO 166528-76 (holotype) | HJB1000133 | Belgium | *Theobromina* | FJ816626, FJ816627 |  | JQ751097 | JQ751130 |  |  |
| *H. aestivale* | HJB10439 | HJB10439 | UK | *Velutipes* | KT218282 | KT217631 | KT217716 |  | KT218048 |  |
| *H. aestivale* | HJB13626 | HJB13626 | Germany | *Velutipes* | KT218447 | KT217675 | KT217767 |  | KT218172 |  |
| *H. aestivale* | S. Poumarat personal collection 10.11.03 | HJB13801 | Spain | *Velutipes* | KT218454 | KT217678 | KT217769 |  | KT218177 |  |
| *H. albidulum* | HJB14702 | HJB14702 | USA | *Velutipes* | MZ782105 | MZ782955 | MZ782923 |  | ON202573 |  |
| *H. albidulum* | HJB16965 | HJB16965 | USA | *Velutipes* | MZ782142 | MZ782954 | MZ782922 |  | ON202572 |  |
| *H. albidulum* | HJB18372 | HJB18372 | USA | *Velutipes* | MZ782147 | MZ782953 | MZ782921 |  | ON202571 |  |
| *H. albidulum* | NYS-F-133.1 (lectotype) | HJB1000451 | USA | *Velutipes* | MN017799 |  |  |  |  |  |
| *H. celatum* | HJB11622 | HJB11622 | Belgium | *velutipes* | KT218356 | KT217651 | KT217739 |  | KT218101 |  |
| *H. celatum* | MAK 08-10021 | HJB12857 | Macedonia | *Velutipes* | KT218413 | KT217664 | KT217752 |  | KT218146 |  |
| *H. celatum* | BR 5020184119676 (holotype) | HJB13621 | Germany | *Velutipes* | KT218446 | KT217674 | KT217766 |  | KT218171 |  |
| *H. citrisporum* | MuOb255034 | HJB15832 | Japan | *Velutipes* | MZ782109 | MZ782971 | MZ782938 |  | ON202574 |  |
| *H. citrisporum* | BR 5020214140236V (holotype check) | HJB15833 | Japan | *Velutipes* | MZ782110 | MZ782972 | MZ782939 |  | ON202575 |  |
| *H. citrisporum* | TNS-F-44560 | HJB16217 | Japan | *Velutipes* | MZ782117 | MZ782970 | MZ782940 |  | ON202576 |  |
| *H. citrisporum* | MuOb295075 | HJB17412 | Japan | *Velutipes* | MZ782145 | MZ782973 | MZ782941 |  | ON202577 |  |
| *H. erebium* | C JV-06-690 | HJB11857 | Denmark | *Velutipes* | KT218373 | KT217657 | KT217745 |  | KT218112 |  |
| *H. erebium* | HJB13620 | HJB13620 | Germany | *Velutipes* | KT218445 | KT217673 | KT217765 |  | KT218170 |  |
| *H. erebium* | LOD IK-H0182 | HJB14180 | Poland | *Velutipes* | KT218463 | KT217682 | KT217773 |  | KT218186 |  |
| *H. erebium* | L 0053546 (holotype) | HJB1000246 | Netherlands | *Velutipes* | KT218481 |  |  |  | KT218197 |  |
| *H. incarnatulum* | HJB10132 | HJB10132 | Estonia | *Velutipes* | KT218239 | KT217606 | KT217695 |  | KT218013 |  |
| *H. incarnatulum* | HJB10139 | HJB10139 | Estonia | *Velutipes* | KT218246 | KT217607 | KT217697 |  | KT218017 |  |
| *H. incarnatulum* | HJB13589 | HJB13589 | Germany | *Velutipes* | KT218441 | KT217672 | KT217764 |  | KT218168 |  |
| *H. incarnatulum* | MICH 10752 (holotype) | HJB1000136 | USA | *Velutipes* | KT218477 |  |  |  | KT218194 |  |
| *H. leucosarx* | HJB9289 | HJB9289 | UK | *Velutipes* | KT218219 | KT217599 | KT217687 |  | KT217999 |  |
| *H. leucosarx* | HJB10140 | HJB10140 | Estonia | *Velutipes* | KT218247 | KT217608 | KT217698 |  | KT218018 |  |
| *H. leucosarx* | HJB11694 | HJB11694 | Belgium | *Velutipes* | KT218361 | KT217652 | KT217740 |  | KT218105 |  |
| *H. leucosarx* | K(M)52712 (lectotype) | HJB1000002 | UK | *Velutipes* | KT218469 |  |  |  |  |  |
| *H. neurophyllum* | D. Lewis personal collection 11907 | HJB15699 | USA | *Velutipes* | ON202544 | ON167814 | ON167859 |  | ON202606 |  |
| *H. neurophyllum* | TLXM AK3782 | HJB16773 | Mexico | *velutipes* | ON202543 |  |  |  | ON202605 |  |
| *H. neurophyllum* | HJB16991 | HJB16991 | USA | *Velutipes* | ON202546 | ON167816 | ON167883 |  | ON202608 |  |
| *H. neurophyllum* | DENA 61424 | HJB17897 | USA | *Velutipes* | ON202545 | ON167815 | ON167892 |  | ON202607 |  |
| *H. neurophyllum* | HJB17981 | HJB17981 | Canada | *Velutipes* | ON202541 | ON167812 | ON167893 |  | ON202603 |  |
| *H. neurophyllum* | HJB18101 | HJB18101 | Canada | *Velutipes* | ON202542 | ON167813 | ON167894 |  | ON202604 |  |
| *H. neurophyllum* | WTU-F-039596 (isotype) | HJB1000558 | USA | *Velutipes* | ON202547 |  |  |  |  |  |
| *H. quercetorum* | HJB10327 | HJB10327 | Italy | *Velutipes* | KT218268 | KT217620 | KT217708 |  | KT218033 |  |
| *H. quercetorum* | HJB13418 | HJB13418 | Spain | *Velutipes* | KT218435 | KT217669 | KT217760 |  | KT218161 |  |
| *H. quercetorum* | HJB13497 | HJB13497 | Cyprus | *Velutipes* | KT218437 | KT217670 | KT217761 |  | KT218164 |  |
| *H. quercetorum* | ROHB 01218 (lectotype) | HJB1000223 | Italy | *Velutipes* | KT218480 |  |  |  | KT218196 |  |
| *H. subconcolor* | HJB11114 | HJB11114 | Swtizerland | *Velutipes* | KT218330 | KT217646 | KT217732 |  | KT218083 |  |
| *H. subconcolor* | HJB11142 | HJB11142 | Switzerland | *Velutipes* | KT218332 | KT217647 | KT217734 |  | KT218085 |  |
| *H. subconcolor* | C-F-8242 | HJB17065 | Greenland | *Velutipes* | MW445847 | MZ782982 | MZ782949 |  | ON202578 |  |
| *H. subconcolor* | LY BR69-12 (holotype) | HJB1000044 | France | *Velutipes* | KT218473 |  |  |  | KT218191 |  |
| *H. velutipes* | C JV-04-551 | HJB10329 | Italy | *Velutipes* | EU570173 | KT217622 | JQ751093 |  | KT218035 |  |
| *H. velutipes* | HJB10350 | HJB10350 | Belgium | *Velutipes* | KT218274 | KT217626 | KT217712 |  | KT218040 |  |
| *H. velutipes* | HJB10588 | HJB10588 | UK | *Velutipes* | KT218294 | KT217635 | KT217719 |  | KT218054 |  |
| *H. velutipes* | HJB11315 | HJB11315 | Portugal | *Velutipes* | KT218342 | KT217649 | KT217737 |  | KT218094 |  |
| *H. velutipes* | HJB11756 | HJB11756 | France | *Velutipes* | KT218364 | KT217653 | KT217741 |  | KT218107 |  |
| *H. velutipes* | HJB11951 | HJB11951 | Svalbard | *Velutipes* | KT218376 | KT217658 | KT217746 |  | KT218114 |  |
| *H. velutipes* | C-F-103582 | HJB16689 | Greenland | *Velutipes* | MW445846 | ON167787 | ON167865 |  | ON202586 |  |
| *H. velutipes* | TLXM HJB16738 | HJB16738 | Mexico | *Velutipes* | ON202567 |  | ON167868 |  | ON202611 |  |
| *H. velutipes* | TLXM AK3749 | HJB16797 | Mexico | *Velutipes* | ON202566 | ON167826 | ON167873 |  | ON202610 |  |
| *H. velutipes* | TLXM HJB16815 | HJB16815 | Mexico | *Velutipes* | ON202568 | ON167827 | ON167878 |  | ON202612 |  |
| *H. velutipes* | TLXM HJB16816 | HJB16816 | Mexico | *Velutipes* | ON202569 | ON167828 | ON167879 |  | ON202613 |  |
| *H. velutipes* | N. Siegel personal collection 3095 | HJB18774 | USA | *Velutipes* | ON202570 | ON167829 | ON167895 |  | ON202614 |  |
